# Serotonin modulation in the male *Aedes aegypti* ear influences hearing

**DOI:** 10.3389/fphys.2022.931567

**Published:** 2022-08-29

**Authors:** Yifeng Y. J. Xu, YuMin M. Loh, Tai-Ting Lee, Takuro S. Ohashi, Matthew P. Su, Azusa Kamikouchi

**Affiliations:** ^1^ Graduate School of Science, Nagoya University, Nagoya, Japan; ^2^ Institute for Advanced Research, Nagoya University, Nagoya, Japan; ^3^ Graduate School of Life Sciences, Tohoku University, Sendai, Japan

**Keywords:** serotonin, *Aedes aegypti*, hearing, mosquito, phonotaxis, neuromodulation, vibrometry, Johnston’s organ

## Abstract

Male *Aedes aegypti* (*Ae. aegypti*) mosquitoes rely on hearing to identify conspecific females for mating, with the male attraction to the sound of flying females (“phonotaxis”) an important behavior in the initial courtship stage. Hearing thus represents a promising target for novel methods of mosquito control, and hearing behaviors (such as male phonotaxis) can be targeted *via* the use of sound traps. These traps unfortunately have proven to be relatively ineffective during field deployment. Shifting the target from hearing behavior to hearing function could therefore offer a novel method of interfering with *Ae. aegypti* mating. Numerous neurotransmitters, including serotonin (5-hydroxytryptamine, or 5-HT) and octopamine, are expressed in the male ear, with modulation of the latter proven to influence the mechanical responses of the ear to sound. The effect of serotonin modulation however remains underexplored despite its significant role in determining many key behaviors and biological processes of animals. Here we investigated the influence of serotonin on the *Ae. aegypti* hearing function and behaviors. Using immunohistochemistry, we found significant expression of serotonin in the male and female *Ae. aegypti* ears. In the male ear, presynaptic sites identified *via* antibody labelling showed only partial overlap with serotonin. Next, we used RT-qPCR to identify and quantify the expression levels of three different serotonin receptor families (5-HT_1_, 5-HT_2_, and 5-HT_7_) in the mosquito heads and ears. Although all receptors were identified in the ears of both sexes, those from the 5-HT_7_ family were significantly more expressed in the ears relative to the heads. We then thoracically injected serotonin-related compounds into the mosquitoes and found a significant, reversible effect of serotonin exposure on the male ear mechanical tuning frequency. Finally, oral administration of a serotonin-synthesis inhibitor altered male phonotaxis. The mosquito serotonergic system and its receptors thus represent interesting targets for novel methods of mosquito, and thus disease, control.

## Introduction

Hundreds of millions of people worldwide are infected with mosquito-borne diseases every year (Abbafati et al., 2020). Diseases transmitted by the yellow fever mosquito *Aedes aegypti* (*Ae. aegypti*) are of particular concern given their rapid global spread and the lack of widely available effective treatments (Bhatt et al., 2013; Kraemer et al., 2019). Strategic use of insecticides thus forms a major component of *Ae. aegypti* control; the recent rapid development of insecticidal resistance has put these methods, and global public health, at risk (Liu, 2015). New methodologies of *Ae. aegypti* control with novel targets are desperately needed.

Male mosquito hearing is one such target due to the importance of hearing during mating (Andrés et al., 2020). Mosquito mating typically occurs in swarms, large male-dominated aggregations which occur only at specific times of day (Clements, 1999). Male *Ae. aegypti* locate conspecific females by listening for the unique sounds females generate during flight. The wing beat frequency (WBF) of these flight tones is particularly important, as it is sexually dimorphic (∼500 Hz in females, ∼750 Hz in males) and thus facilitates rapid identification of flying females (Lapshin, 2012; Staunton et al., 2019). By interfering with this attraction to sound (known as “phonotaxis”), which is sufficiently strong that males show attraction to even artificial female-like sounds, it should be possible to also interfere with mosquito mating behaviors (Johnson and Ritchie, 2016; Andrés et al., 2020).

This has previously been attempted *via* the use of sound traps; speakers playing female flight sounds coupled with a collection device (such as a fan) to contain all attracted males (Staunton et al., 2021). These devices, whilst highly effective in the lab, have failed to demonstrate significant efficacy in the field (Johnson and Ritchie, 2016). Rather than targeting hearing behavior directly therefore, it may be more effective to target hearing function itself.

Male *Ae. aegypti* require a highly sensitive, complex hearing organ to overcome the significant acoustic challenges they face in identifying the small number of females in a male-dominated environment (Somers et al., 2022). The mosquito ear is comprised of two parts—a hairy flagellum which acts as a sound sail, and a Johnston’s organ (JO), the site of auditory mechanotransduction equivalent to a mammalian cochlear (Boo and Richards, 1975b). The resonance frequency at which the male flagellum maximally vibrates matches that of the female mosquito flight sound frequency, enabling males to detect potential mates (Göpfert and Robert, 2001; Jackson and Robert, 2006). Furthermore, the JO itself is incredibly complex. The male *Ae. aegypti* JO contains ∼15,000 neurons, approximately twice as many as the conspecific female JO, making it the largest chordotonal organ in the insect kingdom (Boo and Richards, 1975a; Boo and Richards, 1975b). Uniquely amongst insects, mosquito JOs contain auditory efferents which enable the transmission of information from the brain to the JO, an auditory signature not found in the hearing organ of the fruit-fly, *Drosophila melanogaster* (*D. melanogaster*) (Kamikouchi et al., 2010; Andrés et al., 2016; Su et al., 2018).

The mechanical frequency tuning of the male flagellar ear relies in part on the neurotransmitter systems found within the JO (Andrés et al., 2016). Injection of neurotransmitter receptor agonists/antagonists, such as octopamine, into male mosquitoes can influence their flagellar ear mechanical tuning frequency, potentially altering their hearing behaviors. The role of serotonin (5-hydroxytryptamine, or 5-HT) however has gone relatively under researched despite its previous identification in male *Ae. aegypti* and *Culex quinquefasciatus* JOs (Siju et al., 2008; Andrés et al., 2016).

Biogenic amines bring about important changes in various physiological functions and behaviors of living organisms by promoting chemical cross-talk between neurons, thereby modulating the excitability of the neural network (Destexhe and Marder, 2004; Nadim and Bucher, 2014). Biogenic amines exert their effects by binding to G-protein-coupled receptors (GPCRs). Depending on the class of G-protein associated with the GPCR, a given neurotransmitter can elicit distinct effects on neurons, such as inhibition or stimulation, conferring an extra layer of flexibility to the structurally restrictive neural network (Kringelbach et al., 2020).

Biogenic amines such as serotonin, octopamine/tyramine and dopamine, have been shown to influence various insect behaviors including aggregation, mating, feeding, and sleeping (Anstey et al., 2009; Fuchs et al., 2014; Ngai et al., 2019). Serotonin itself has also been directly linked to behavioral transitions from solitary to swarming in desert locusts and is associated with the regulation of circadian behaviors across multiple species (Yuan et al., 2005; Anstey et al., 2009; Morin, 2009).

In insects, there are three major families of serotonin receptors, namely 5-HT_1_, 5-HT_2_, and 5-HT_7_, with each receptor capable of activating distinct intracellular transduction pathways (Fuchs et al., 2014; Ngai et al., 2019). Agonizing or antagonizing these serotonin receptors *via* pharmacological approaches has been shown to affect, amongst other behaviors, *Ae. aegypti* flight performance (Ngai et al., 2019). This is highly relevant in the context of mosquito acoustic communication, as flight plays a key role in the hearing-mediated mating behavior of mosquitoes, with even relatively small changes in the frequency of flight sounds resulting in significant changes in the male responses (Andrés et al., 2020; Su et al., 2020). However, it remains unknown whether manipulation of the serotonergic system in *Ae. aegypti* could alter their hearing system. It is also unclear which, if any, serotonin receptors are expressed in their JO.

Here, we investigated the role of serotonin modulation in determining the *Ae. aegypti* hearing function and behavior. First, we found clear serotonergic innervation of the JO in both sexes. We further identified some, albeit limited, overlap between presynaptic sites and serotonin in male JOs, with considerably more overlap found in female JOs. Using RT-qPCR, we quantified relative expression of six serotonin receptors in the JOs, heads, and bodies of males and females, with receptors of the 5-HT_7_ subfamily having significantly higher expression levels in JOs compared to heads in both sexes.

Thoracic injection of serotonin significantly and sizably altered male hearing function, with changes in female hearing function small but also statistically significant. This change in hearing function in males was partially recoverable *via* injection of a non-selective serotonin receptor antagonist. Finally, by oral administration of a pharmacological compound that altered serotonin levels, we further found that manipulation of the serotonergic system can affect male hearing behaviors. Our findings reveal that the serotonergic system of male *Ae. aegypti* plays a key role in influencing their hearing system, making it a suitable target for vector control.

## Materials and methods

### Mosquito rearing

*Ae. aegypti* (Liverpool strain) mosquitoes were reared in a 12 h:12 h light–dark cycle at 26–28°C and 60%–70% relative humidity. Adults were provided with a constant source of 10% glucose mixture. Horse blood feeding when necessary was conducted using an Orinno blood feeding system (Orinno Technology Pte Ltd., Singapore).

All mosquitoes used for experiments were between 4 and 9 days old. At least 2 days prior to all experiments, mosquitoes were transferred into light, temperature and humidity-controlled incubators set at 28°C and 60% relative humidity. Light stimulus, provided *via* LED dimmable lights (Hipargero LED), followed a pattern of 1 h dawn (constant increase of light to maximum), 11 h constant light, 1 h dusk (constant decrease of light to minimum), and 11 h constant dark.

### JO immunohistochemistry

JO section immunohistochemistry (IHC) followed previously published protocols (Andrés et al., 2016; Su et al., 2018). Whole mosquito heads were removed on ice and fixed with 4% paraformaldehyde (PFA) in Phosphate-buffered saline (PBS) containing 0.25% Triton X-100 (PBT) for 1 h. Heads were embedded in 6% albumin, and the albumin blocks were allowed to solidify at 4°C for 10 min before overnight fixation in 4% PFA at 4°C. Samples were washed in 100% methanol for 10 min, then washed in PBS for 30 min and sectioned in 40 µm increments using a vibratome (Leica VT1200S).

Sections were washed three times in 0.5% PBT and blocked in 10% normal goat serum (NGS) (Vector Laboratories, Inc.)/0.5% PBT for 1 h at room temperature. Primary antibodies in 10% NGS were then added, and the samples were incubated overnight at 4°C.

After three washes with 0.5% PBT, samples were incubated with secondary antibodies in 10% NGS for 2 h at room temperature. Following this, samples were washed three times with 0.5% PBT before a final PBS wash. Samples were mounted on slide glasses and imaged using a laser-scanning confocal microscope (FV3000, Olympus) equipped with a 20× air objective (UPlanSApo, NA = 0.75) and silicone-oil immersion 30× or 60× Plan-Apochromat objective lens (UPlanSApo, NA = 1.05 and 1.3, respectively).

Primary antibodies used were anti-SYNORF1 3C11 monoclonal antibody (AB_528479, 1:30, Developmental Studies Hybridoma Bank (DSHB), University of Iowa), anti-bruchpilot (nc82, AB_2314866, 1:20, DSHB), anti-serotonin monoclonal antibody (MA5-12111, 1:40, ThermoFisher) and anti-serotonin polyclonal antibody (20080, 1:300, ImmunoStar). Dextran 555 (D3308, MW 3000, ThermoFisher) was used for anterograde tracing. The secondary antibodies used were Alexa Fluor 488-conjugated anti-mouse IgG (#A-11029, 1:300, ThermoFisher), Alexa Fluor 647-conjugated anti-rat IgG (#A-21245; 1:300; ThermoFisher) and anti-horseradish peroxidase (anti-HRP, AB_2338959, 1:100, Jackson Immuno Research).

### JO width measurement

Male and female JO images containing HRP signals were analyzed in ImageJ (version 1.53q, National Institutes of Health, RRID: SCR_003070). The width of each JO was taken to be the longest distance between two sides of the JO outer somata. In total, 10 male and 10 female images were included in the analysis.

### RT-qPCR and RT-PCR

Groups of virgin male or virgin (non-blood fed) female mosquitoes were flash-frozen in liquid nitrogen and stored in a −80°C freezer. Mosquitoes were collected at Zeitgeber time 12 (ZT 12) of their entrainment regime, corresponding to dusk. Tissues were then dissected on ice in RNAiso Plus (#9109, Takara Bio Inc.), with JO (the second antennal segment with flagellum removed), head (lacking both antennae and mouthparts), and body (including legs and wings) tissue being collected.

Dissected samples were homogenized (Handheld Homogenizer, BT LabSystems) in RNAiso Plus (#9109, Takara Bio Inc.). Additional RNAiso Plus was added to the lysed samples to make up a final volume of 1 ml. The samples were then inverted several times prior to incubation at room temperature for 5 min. 200 µl chloroform (Kanto Chemical Co., Inc.) was added to each sample and mixed well before incubating at room temperature for 15 min. Samples were then centrifuged at 12,000 × g for 15 min at 4°C before adding 0.5 ml 2-propanol (Sigma). Samples were stored at −20°C for 30 min.

Following centrifugation at 12,000 × g for 10 min at 4°C, the supernatant was discarded, and the RNA pellet was washed with 1 ml of freshly prepared 75% ethanol (Sigma) solution. Samples were centrifuged at 7,500 × g at 4°C for 5 min. The ethanol wash cycle was performed twice before the RNA pellet was dissolved in Nuclease Free Water (Invitrogen). RNA sample quality was confirmed using a Nanodrop (ThermoFisher Scientific) and samples were stored in a −80°C freezer.

For RT-qPCR experiments, RNA was reverse transcribed (ReverTra Ace^TM^qPCR RT Master Mix with gDNA Remover, FSQ-301, TOYOBO) and qPCR mixes prepared (THUNDERBIRD^TM^SYBR^®^qPCR Mix, QPS-201, TOYOBO). The housekeeping gene *ribosomal protein S7* (*rps7*) was used as the internal control. Serotonin receptor primers were designed using the NCBI primer design tool (Table 1). For each qPCR 96-well plate (Bio-Rad), three technical repeats for each sample and primer were tested, with the median of these technical values being used for analyses. In total, seven repeats were conducted for each tissue type for both males and females.

**TABLE 1:** Primer design for RT-qPCR/RT-PCR.

Gene	Gene ID	Primer direction	Sequence	Primer length (bp)	Amplicon length (bp)
5-HT_1A_	AAEL008360	Forward	GTG​GGT​TCG​GCT​CTT​ACC​AA	20	121
		Reverse	TGA​TGT​TGT​CGT​CGC​TCA​CA	20	
5-HT_1B_	AAEL017272	Forward	CTT​CTG​GTG​GCA​TGT​CTC​GT	20	146
		Reverse	GCT​ATC​GCC​ACC​AAA​TGC​AG	20	
5-HT_2A_	AAEL019804	Forward	AAC​CGG​TGT​CGG​AAC​TTC​TC	20	196
		Reverse	TAG​AAA​GTG​AAC​CGA​CCG​GC	20	
5-HT_2B_	AAEL019805	Forward	AAC​CAG​GCA​GCG​AAC​GAT​TA	20	132
		Reverse	GGT​TCC​ATC​CCT​GTC​GTT​GT	20	
5-HT_7A_	AAEL027242	Forward	TAC​ATC​ACT​GCC​TGC​CCT​TG	20	75
		Reverse	CCA​CCG​AGT​TCG​TCG​AGT​AG	20	
5-HT_7B_	AAEL025125	Forward	GTC​GCC​CCA​CCA​AAA​GAA​AC	20	163
		Reverse	GGA​TGA​GAG​GGT​GGG​GTA​GT	20	
ribosomal protein S7 (rps7)	AAEL009496	Forward	ATG​GTT​TTC​GGA​TCA​AAG​G	19	501
		Reverse	CTT​GTG​TTC​AAT​GGT​GGT​CTG	21	
tryptophan hydroxylase (TPH)	AAEL017120	Forward	GAA​GAG​ATC​AAG​ACA​TGG​GGC​A	22	392
		Reverse	GCA​TGT​TGT​AGT​TCG​GCC​AC	20	
serotonin transporter (SERT)	AAEL005581	Forward	AGT​TTT​CTG​GCG​GGA​TTC​GT	20	347
		Reverse	AGG​TAC​ACA​CCG​CCA​TAA​GT	20	

For RT-PCR experiments, following reverse transcription, the cDNA was then used to run PCR (PrimeSTAR^®^ Max DNA Polymerase) with a thermal cycler program of 35 cycles (Bio-Rad T100). The PCR products were analyzed using 2% agarose gel electrophoresis, including staining with DNA-staining fluorescent dye (WSE-7130 EzFluoroStain DNA, ATTO) followed by imaging using a Transilluminator (Daihan Scientific, WUV-M20).

### Phylogenetic tree construction

Predicted protein sequences of putative serotonin receptors for *D. melanogaster*, *Ae. aegypti* and *Anopheles gambiae* (*An. gambiae*) were downloaded from VectorBase (Giraldo-Calderón et al., 2022). Using MEGA software version 11 (Tamura et al., 2021), protein sequences were first aligned in MUltiple Sequence Comparison by Log- Expectation (MUSCLE) (Madeira et al., 2022). To test the reliability of the constructed phylogenetic tree, tree nodes were then tested using Maximum Likelihood analysis with 1,000-fold bootstrap replicates. Bootstrap values were displayed as numbers above each node of a branch. Branch length (scale bar: 0.5 units) represents the number of amino acid substitutions per sequence site. The *Ae. aegypti norepinephrine transporter* (AAEL005581) was selected as an outgroup. Accession numbers for receptors from each species are listed in Table 2.

**TABLE 2:** Accession numbers for phylogenetic tree.

Species	Accession number	Receptor family
*D*. *melanogaster*	FBgn0004168	5-HT_1A_
*D*. *melanogaster*	FBgn0263116	5-HT_1B_
*Ae. aegypti*	AAEL008360	5-HT_1A_
*Ae. aegypti*	AAEL017272	5-HT_1B_
*An. gambiae*	AGAP007136	5-HT_1A_
*An. gambiae*	AGAP011481	5-HT_1B_
*An. gambiae*	AGAP002519	5-HT_1C_
*D*. *melanogaster*	FBgn0087012	5-HT_2A_
*D*. *melanogaster*	FBgn0261929	5-HT_2B_
*Ae. aegypti*	AAEL019804	5-HT_2A_
*Ae. aegypti*	AAEL019805	5-HT_2B_
*An. gambiae*	AGAP002232	5-HT_2A_
*An. gambiae*	AGAP002229	5-HT_2B_
*D*. *melanogaster*	FBgn0004573	5-HT_7_
*Ae. aegypti*	AAEL025125	5-HT_7A_
*Ae. aegypti*	AAEL027242	5-HT_7B_
*An. gambiae*	AGAP004223	5-HT_7A_
*An. gambiae*	AGAP004222	5-HT_7B_

### Laser Doppler vibrometry recordings: Mosquito preparation

All recordings were conducted within the 3 h prior to dusk at 28°C. Preparation of mosquitoes followed previously published protocols (Su et al., 2018). Mosquitoes were sedated on ice, then glued to a small plastic rod (H-13, Narishige Instruments, Japan). Glue (Norland Products Inc., 81) was minimally applied to the body to avoid hindering flagellar movement or obstructing major spiracles. After application of glue, only the right flagellum was free to move.

The rod was held securely by a micromanipulator (MM-3, Narishige Instruments, Japan) placed upon a vibration isolation table. Mosquitoes faced the laser directly and were positioned such that the flagellum was at a 90° angle to the vibrometer. For male mosquitoes, the laser focal point was chosen to be on the second flagellomere from the tip, whilst for females the third flagellomere from the tip was chosen.

Two types of flagellar vibration were recorded; so-called “free fluctuations,” where no stimulation was provided to the flagellum which thus moves as a result of Brownian noise, and white noise recordings, in which a 1–2,000 Hz (70 dB SPL) broadband white noise stimulus (produced using the Audacity software, version 3.0.3, The Audacity team) was played to the mosquito using a speaker (85 mm in diameter, FF85WK, Fostex). All recordings were made using a laser Doppler vibrometer (Vibroflex, Polytec) and data were collected using the Vibsoft software (Polytec).

### Laser Doppler vibrometry recordings: Compound injection

5 or 25 mM serotonin (5-HT) (50-67-9, Sigma) and 5 mM methiothepin mesylate salt (74611-28-2, Sigma) solutions were prepared from 25 mM stock solutions. 25 mM alpha-Methyl-DL-tryptophan (AMTP; 153-91-3, Sigma) and 25 mM 5-Hydroxy-L-tryptophan (5-HTP; 4350-09-8, Sigma) solutions were diluted from 50 mM stock solution. All stock solutions were prepared using Ringer solution (5 M NaCl, 1 M KCl, 1 M CaCl_2_, 1 M MgCl_2_·6H_2_O 1 M NaHCO_3_, 1 M NaH_2_PO_4_, 1 M Sucrose, 0.5 M Trehalose·2H_2_O, and 1 M Hepes-NaOH dissolved in distilled water; pH 7.5; 265 mOsm) (Wang et al., 2003; Lai et al., 2012). Ringer solution alone was used for control injections. Sharpened glass microcapillaries (G1, Narishige) were prepared using a puller (PC-10, Narishige) before being filled with injection solution immediately prior to injections.

After baseline recordings of flagellar movement were completed (3 measurements over a 5 min period), microcapillaries were inserted directly into the mounted mosquito thorax. Solution was then injected so as to flood the whole body.

For single injection experiments, recordings of flagellar movement were made every 2–3 min over a 35 min period to monitor frequency changes (ΔFrequency = post-injection frequency at specific timepoint—baseline frequency) in the flagellar function.

For two injection experiments, the second injection occurred 10 min after the initial serotonin injection. Measurements following the initial serotonin injection were thus recorded every 2–3 min over 10 min, whilst measurements following the second injection were recorded every 2–3 min over the following 35 min.

Total sample sizes were:

Male Ringer injection = 10
Male 25 mM AMTP injection = 15
Male 5 mM serotonin injection = 11
Male 25 mM serotonin injection = 10
Male 25 mM 5-HTP injection = 11
Male 25 mM serotonin and Ringer injection = 10
Male 25 mM serotonin and 5 mM methiothepin injection = 10
Female Ringer injection = 10
Female 5 mM serotonin injection = 10

### Laser Doppler vibrometry recordings: Data analysis

Fast Fourier transforms of flagellar velocity values obtained from white noise vibrometry recordings were calculated using the Vibsoft Polytec software for frequencies between 1 Hz and 10 kHz. Values below 125 Hz were found to contain significant noise and were thus not included in analyses.

The nlm package (version 4.3.0) in R software (version 4.1.1) (R Core Team, 2021) was used to fit the below forced damped harmonic oscillator function, defined as in a previous paper (Göpfert et al., 2005) to velocity values for frequencies between 125 and 1,000 Hz:

X˙(ω)= F0mω2. ((ω02− ω2)2+(ω.ω0Q)2)

Where F_0_ is the external force strength, m is the flagellar apparent mass, ω is the angular frequency, ω_0_ is the natural angular frequency and Q is the quality factor (with Q = mω_0_/γ, with γ being the damping constant).

This function fit then allowed for estimation of the natural angular frequency (ω_0_) for each recording, which was then used to calculate the flagellar best frequency, f_0_ (with f_0_ = ω_0_/2π).

### Phonotaxis assay

Male *Ae. aegypti* were aspirated into cages (4M3030, Bugdorm) in groups of 30 and entrained in incubators under the aforementioned light entrainment paradigm. Cages were positioned such that a speaker (25 cm in diameter, FF225WK, Fostex) placed next to the cage served to provide acoustic stimulation. A camera (DMK33UP1300, ARGO) with a lens (LM5JC1M, Kowa) was placed opposite the speaker to enable recording of male phonotactic response to tone playback. Cotton wool soaked in 10% glucose solution was provided as a food source.

After a full-day of entrainment, the phonotactic response of males was tested for the first time (Day 0 in Figure 4A). Males were exposed to pure tone stimuli in a 1 min on, 1 min off fashion during dusk (ZT 12); for comparative purposes, some phonotaxis experiments were also conducted shortly after dawn (ZT 0.5). Pure tone stimuli in the frequency range of 350—750 Hz (25 Hz intervals) were provided in a quasi-random order each time. Speaker output was calibrated such that each tone was played at the same intensity (70 dB SPL).

Immediately after the end of the stimulus period, glucose food was removed from the incubator, and males were starved for 21 h. Cotton wool soaked in either 10% Ringer in 10% glucose solution (control solution) or 5 mM AMTP (prepared from a 50 mM stock solution) in 10% glucose solution was provided to the males. Males were allowed to feed for 3 h on the compound-doped glucose food before their responses to sound were tested for the second time during dusk (Day 1 in Figure 4A). For cages that were fed with 5 mM AMTP in 10% glucose solution, immediately after the second round of testing the AMTP-doped solution was replaced with fresh Ringer-doped solution. These groups of males were then subjected to another round of testing on the following day to check for signs of phonotactic recovery (Day 2 in Figure 4A).

Video playback was analyzed manually by counting the number of males which landed on the cage netting directly next to the speaker during tone playback. Prior to the onset of each tone, the number of males that had already landed on top of the netting in front of the speaker was counted and subtracted from the number of males which then proceeded to land on the netting during playback. Male response to each tone was blindly scored without knowing the tone order provided during each test. To account for variations in the overall activity level between different repeats (i.e., across different cages), the phonotactic response of males in each treated-condition/day was normalized to the largest number of responders within the same repeat.

In total, ten cages were exposed to Ringer-doped glucose food (control), and eleven cages were exposed to 5 mM AMTP-doped glucose food. Of these eleven cages, nine were provided with Ringer-doped food and tested for a further day to check for potential recovery of hearing behaviors.

### ELISA assay

Groups of 20 male mosquitoes were provided with 10% glucose food and entrained for 2 days in incubators to the aforementioned entrainment paradigm. At dusk on the third day, glucose food was removed and the males were starved overnight until 3 h prior to dusk of the next day. Males were then provided with 10% glucose food doped with both a food coloring dye (to allow for confirmation of which males had consumed doped food) and either a control Ringer solution, 5 mM AMTP in Ringer solution or 25 mM AMTP in Ringer solution.

Males were allowed to feed for 3 h before being flash frozen in liquid nitrogen. Mosquitoes were screened for food consumption *via* visual confirmation of the presence of dye in their abdomen, and 10 doped food-consuming males from each group were used for testing. In total, three repeats for each food type were conducted.

ELISA assays utilized an Serotonin ELISA kit (BA-E-5900R, Immusmol) and followed the manufacturer’s protocol. Samples were first homogenized on ice in Diluent buffer followed by centrifugation at 15,000 g for 10 min at 4°C. The supernatant (diluted to a final concentration of 0.1×) was then used in the subsequent ELISA steps. A plate reader (EnSpire Multimode Plate Reader, PerkinElmer Inc.) was utilized to quantify the absorbance of samples in both 450 and 620 nm wavelength ranges, with the latter serving as a reference. Testing of standard solutions with known absolute serotonin concentrations allowed for creation of fitting curve, enabling estimation of serotonin concentrations for all tested samples (in ng/ml). These estimates were then multiplied by a factor of 10 to adjust for the original tenfold dilution.

### Statistical analysis

*p* < 0.05 (prior to Bonferroni correction) was set as the significance level for all statistical tests conducted.

A Wilcoxon rank sum test was used to test for significant differences between male and female JO diameters.

For all qPCR experiments, median *rps7* values were used to calculate DeltaCt (ΔCt) values for each gene. DeltaDeltaCt (ΔΔCt) values were then calculated within a repeat using head samples as references; all head ΔΔCt values were thus equal to 0. Repeat ANOVA tests (with correction for multiple comparisons) were used for each sex to test for significant differences in expression within each set of genes between different tissues.

To evaluate the significance of changes in flagellar ear mechanical tuning frequency after a single injection, loess curves were fit to time-series plots (lasting 35 min) of extracted frequencies for individual mosquitoes by using the loess function from the stats package (version 4.1.2; R Core Team). The loess curve was fit such that estimated frequencies could be extracted at one-minute intervals. The median difference in ear mechanical tuning frequency (median ΔFrequency) was calculated by subtracting the baseline tuning frequency prior to injection from each of these estimated frequencies (i.e., estimated frequencies between 1 min and 35 min after injection at 1 min intervals), and then finding the median of these values. ART ANOVA tests were then run to check for significant differences between pre- and post-injection states for each group within a sex.

To analyze two injection experiments (25 mM serotonin injection followed by a further injection), a loess curve was fit to data recorded within 10 min after serotonin injection and the median ΔFrequency extracted. A loess curve was then fit to frequency values extracted from data recorded within the 30 min following the second injection at 10 min; the minimum value from this fit was then estimated. This allowed for calculation of Min. ΔFrequency between ear mechanical tuning frequency after serotonin injection and the estimated minimum value following the second injection. ART ANOVA tests were then run to check for significant differences between after serotonin and after second injection states.

The area under the curve (AUC) of each phonotactic profile was estimated using the AUC function from the DescTools package (version 0.99.44) (Signorell et al., 2021). Wilcoxon signed-rank tests were then used to test for significant differences in the AUC before and after doped food exposure.

Loess curve was fitted to the individual phonotactic profiles using geom_smooth function from the ggplot2 package (Wickham, 2016) in R. The phonotactic response range (upper boundary frequency and lower boundary frequency) of each loess fitted phonotactic profile was estimated by first defining a minimum response threshold of 25% of that of the peak response frequency. For the left tail of the curve, the lower boundary frequency is the last frequency before which the response would surpass the minimum respond threshold. For the right tail of the curve, the upper boundary frequency reflects the first frequency beyond which the response would fall below the minimum response threshold. Wilcoxon signed-rank tests were then used to test for significant differences in the upper frequency boundaries before and after doped food exposure.

## Results

### Serotonin is expressed, and has similar expression patterns, in male and female *Ae. aegypti* ears

**FIGURE 1:**
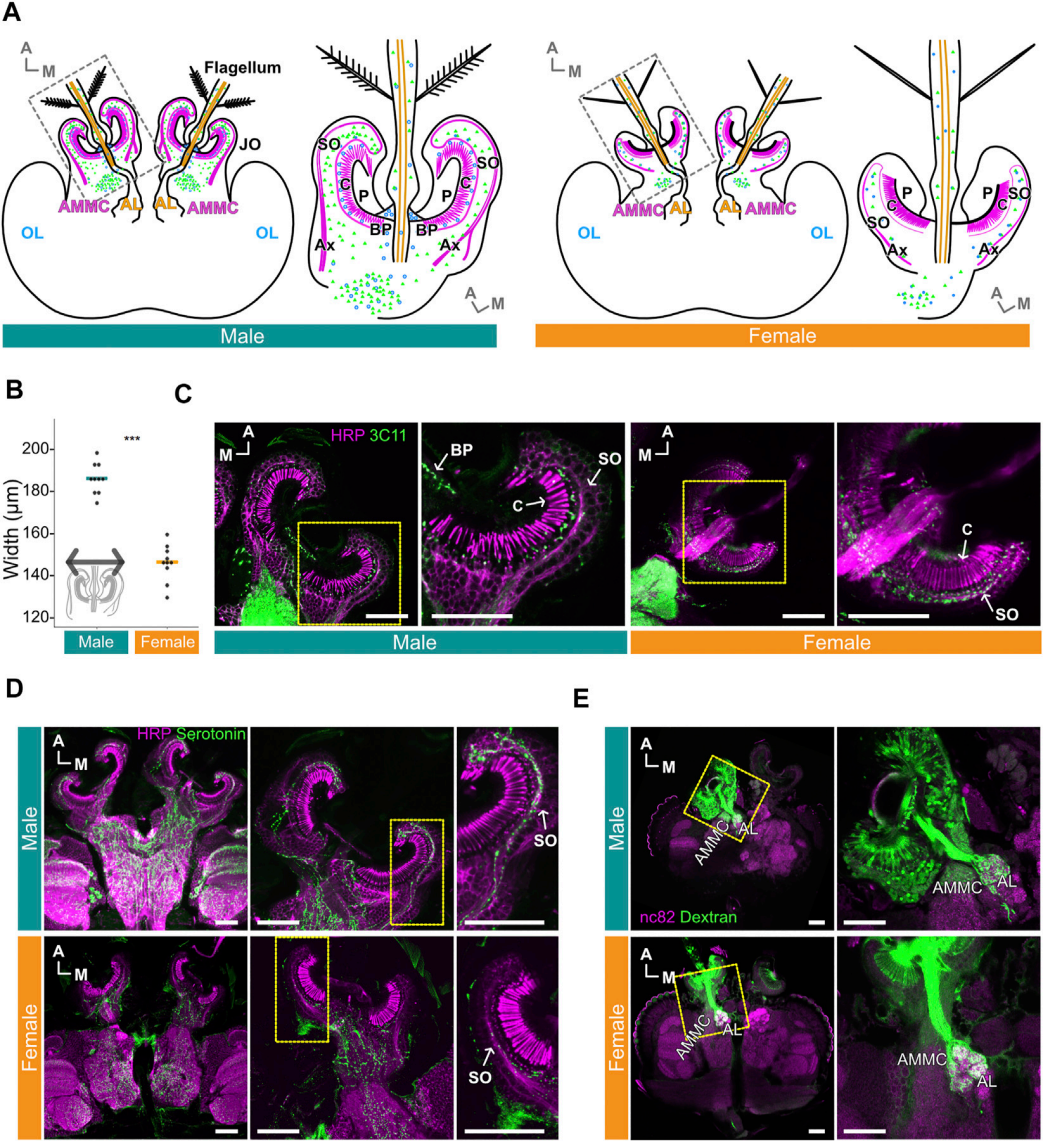
Serotonin is distributed throughout male and female *Ae. aegypti flagellar* ears. **(A)** Schematic diagrams of male (left) and female (right) brains and ears (JO and flagellum). Magenta and orange represent JO neurons and flagellar neurons in the ear, respectively. Green and blue dots represent serotonin and presynaptic sites, respectively. AL, Antennal lobe; AMMC, Antennal mechanosensory and motor center; Ax, axons of JO neurons; BP, basal plate; C, cilia of JO neurons; OL, optic lobe; P, prong; SO, somata array of JO neurons. **(B)** Diameters of male and female JOs (in μm). Dots represent individual diameter values, solid bars represent medians for each group. Inset, representation of length used to define diameter. **(C)** Presynaptic sites in JOs. Sections of male (left) and female (right) JOs are shown anti-horseradish peroxidase (HRP) staining (magenta) allows for visualizing neurons, anti-SYNORF1 (3C11) staining (green) labels presynaptic sites. Yellow frames indicate the positions for the right panels. **(D)** Serotonin signal in male and female brains and JOs. HRP staining (magenta) allows for visualizing neurons, serotonin staining (green) shows location of serotonin. Yellow frames in the middle panels indicate the positions for the right panels. **(E)** Projection patterns of JO and flagellar neurons from the ear to the brain in male (top) and female (bottom) as visualized by anterograde Dextran staining. anti-bruchpilot (nc82) staining (magenta) allows for visualizing neurons, Dextran dye staining (green) traces JO and flagellar neurons from the ear to the brain. Yellow frames indicate the positions for the right panels. **(A–E)** A, anterior; M, medial. Horizontal views of the brain and JO. **(C–E)** Scale bar = 50 μm.

Male and female *Ae. aegypti* JO*s* show extreme sexual dimorphisms in terms of overall size, JO neuron count and the distribution patterns of presynaptic sites (Boo and Richards, 1975a; Andrés et al., 2020) (Figure 1A). The width of the male JO is approximately 40 μm bigger than the female JO (*p* < 0.001, Wilcoxon rank sum test; Figure 1B). Staining of the *Ae. aegypti* ears with the presynaptic marker 3C11 (anti- SYNORF1) further demonstrates these dimorphisms. Though the 3C11 signals were almost entirely restricted to the somata of JO neurons in females, male sections show strong expression in the space between the somata and cilia, as well as limited expression at the basal plate and in the somata (Figures 1A,C). These findings suggest distinct distributions of presynaptic sites in the male and female JOs.

Despite this, serotonin is broadly expressed in the somata of JO neurons in both males and females, though seemingly more abundant in males (Figure 1D). Furthermore, some serotonin was identified in, and at the base of, the flagellum in both sexes (Supplementary Figure S1A). Despite the abundant expression of serotonin in the male JO, potential overlap between presynaptic sites (as visualized by 3C11) and serotonin itself appeared to be minimal in males as compared to the females (Supplementary Figure S1B).

To examine if there are any fundamental sexual differences in the projection pattern of JO neurons to the brain, we adopted an anterograde dye-filling approach to visualize these projection patterns. Here, we define flagellar neurons as neurons housed in the flagellum itself and flagellar axons as nerve bundles descending from these neurons. JO neurons are neurons whose somata are located in the JO and are structurally separated from the flagellar neurons. Our dye-filling experiment suggests a conserved projection pattern of flagellar neurons and JO neurons to the brain in both sexes (Figure 1E). In males and females, we found that the flagellar neurons project their nerves to the antennal lobe (AL), which is characterized by its glomeruli-rich structure. The axons of JO neurons, on the other hand, extend to a neuropile diagonally lateral to the AL (Figure 1E), which is known to be the location of the antennal mechanosensory and motor center (AMMC) in the mosquito brain, as well as other insect species (Kamikouchi et al., 2006; Mosquito Brain Browser, 2022). We thus confirmed that the neuropil innervated by the axons of JO neurons is the AMMC.

### Serotonin receptors are expressed in male and female *Ae. aegypti* JOs

Though the exact number of receptor members in each serotonin receptor subfamily varies across insect species, the importance of serotonin in determining insect behaviors (such as locomotion and aggregation) remains consistent (Kamhi et al., 2017). In *Ae. aegypti*, six putative serotonin receptors in total have been identified, with each receptor subfamily (5-HT_1_, 5-HT_2_, and 5-HT_7_) comprising two receptor members (denoted here as receptor A and B) (Ngai et al., 2019). A full phylogenetic tree built from the protein sequence alignment of serotonin receptors of *Ae. aegypti*, *D. melanogaster* and *An. gambiae* receptors, is shown in Supplementary Figure S2.

**FIGURE 2:**
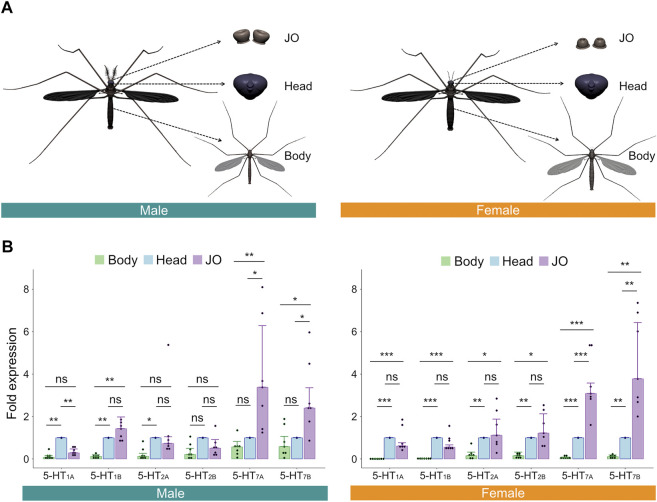
Serotonin receptors are expressed in male and female *Ae. aegypti* JOs. **(A)** Diagrams of tissues dissected and tested *via* RT-qPCR. **(B)** Fold expression of each of the six serotonin receptors in male (left) and female (right) bodies, heads, and JOs. Seven repeats were conducted for each tissue in both sexes. The bar graph shows the median with an SEM error bar. Each dot represents the fold expression of each repeat. Fold expressions of body (green) and JO (purple) were normalized by that of the head (blue). ns, *p* > 0.05; *, *p* ≤ 0.05; **, *p* ≤ 0.01; ***, *p* ≤ 0.001.

To confirm the expression levels of these serotonin receptors in male and female JOs, we dissected virgin, non-blood fed female and virgin male mosquitoes collected at ZT 12 [corresponding to dusk and assumed to be the peak swarming time of mosquitoes (Manoukis et al., 2009; Somers et al., 2022)] and extracted the RNA from three distinct tissues—JOs (second antennal segments), heads and whole bodies (Figure 2A). We then performed RT-qPCR to compare the relative expression of each receptor in the JO and body, as compared to the head.

For all members of the 5-HT_1_ and 5-HT_2_ receptor subfamilies, expression levels in the JO were not significantly different from levels in the head for females (*p* > 0.05, Repeat ANOVA; all values provided in Supplementary Table S1; Figure 2B, right). For males, expression of 5-HT_1B_ and both members of the 5-HT_2_ family were not significantly different between JOs and heads (*p* > 0.05, Repeat ANOVA; all values provided in Supplementary Table S2; Figure 2B, left), though 5-HT_1A_ was significantly more expressed in heads than JOs (*p* < 0.01, Repeat ANOVA). Interestingly, in both sexes, we identified significantly higher expression levels of both members of the 5-HT_7_ subfamily in the JOs, as compared to the head (*p* < 0.05 for all comparisons; Repeat ANOVA; all values provided in Supplementary Tables S1, S2; Figure 2B). 5-HT_7_ receptors thus appear uniquely over-expressed in the JOs of males and females as compared to other tissues.

Finally, using RT-PCR, we confirmed the expression of two serotonergic neuronal markers, *tryptophan hydroxylase* (TPH) (one of the intermediate enzymes essential for serotonin synthesis) and *serotonin transporter* (SERT) (the transporter involved in the reuptake of serotonin from the synaptic cleft into the serotonin-releasing presynaptic neuron) in male and female bodies, heads and JOs (Supplementary Figures S2B,C).

### Male hearing function is significantly and sizably altered after serotonin injection, with this alteration partially recoverable *via* injection of non-selective antagonists

**FIGURE 3:**
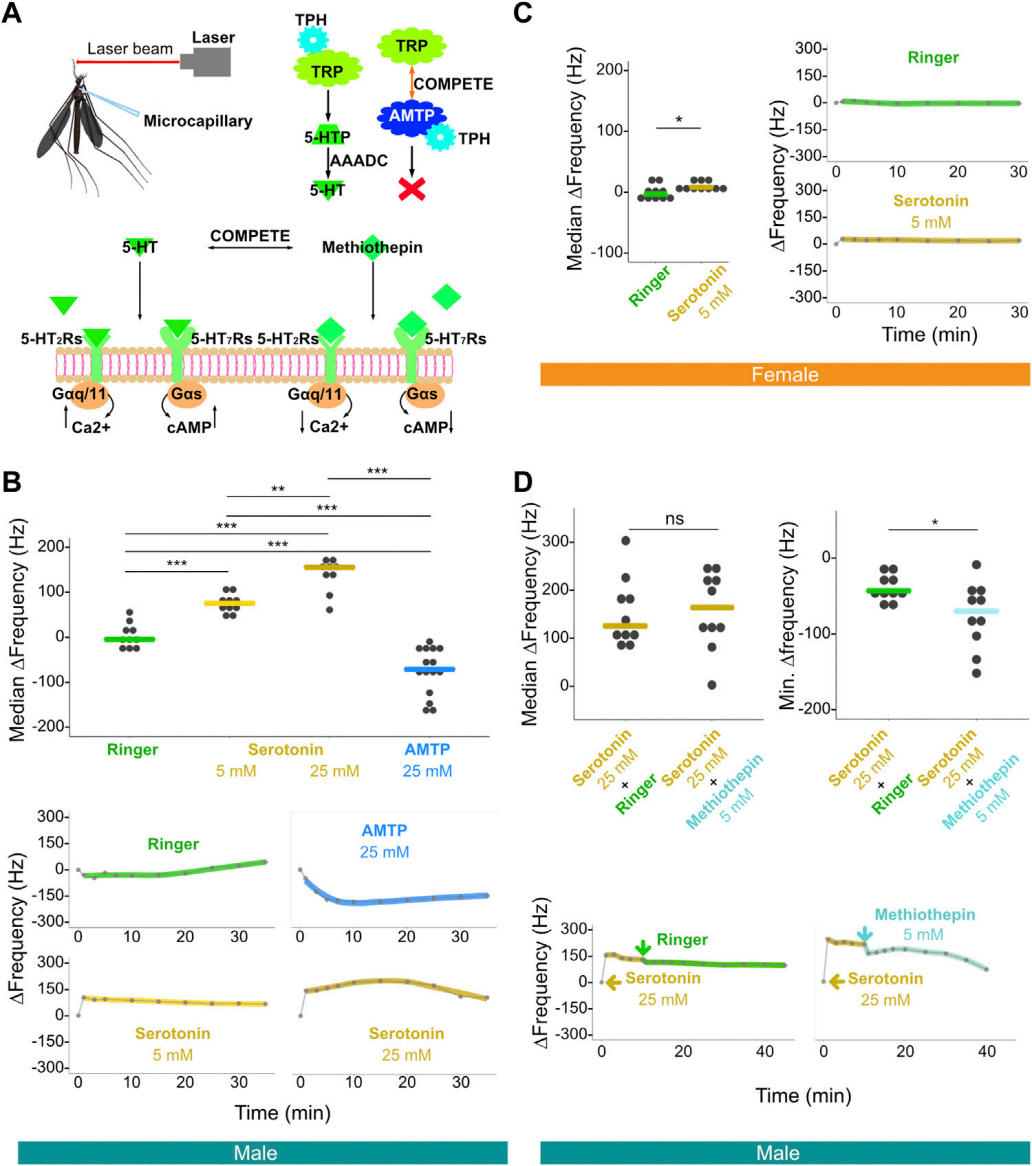
Injection of serotonin-modulating compounds influences male, and to a lesser extent female, flagellar ear mechanical tuning frequency. **(A)** Diagram of laser experimental setup. This illustration includes microcapillary used for injections (upper left), alpha-methyltryptophan (AMTP) working mechanism (upper right) and serotonin receptor functional properties (bottom); 5-HT, serotonin; 5-HT_2_Rs, 5-HT_2_ receptors; 5-HT_7_Rs, 5-HT_7_ receptors; 5-HTP, 5-hydroxytryptophan; AAADC, aromatic L-amino acid decarboxylase; AMTP, alpha-methyltryptophan; TPH, tryptophan hydroxylase; TRP, tryptophan. **(B)** Median ΔFrequency data for Ringer, 5 mM serotonin, 25 mM serotonin, or 25 mM AMTP male single injection groups (top). Significant differences identified *via* ART ANOVA between groups are labelled. Dots represent median ΔFrequency for individuals. Bars represent the median of the median ΔFrequency for each group. Representative plots from individuals that show the time series of changes in flagellar ear mechanical tuning frequency (ΔFrequency) over 35 min (bottom) following Ringer (top left), 25 mM AMTP (top right), 5 mM serotonin (bottom left) or 25 mM serotonin (bottom right) injection. Injection occurred immediately after the first recording at zero min. Dots represent frequency values extracted from collected data. Solid lines in the bottom panels represent loess fits to these data points. **(C)** Median ΔFrequency data (left) for Ringer (left) or 5 mM serotonin (right) female single injection groups. Significant differences identified *via* ART ANOVA between groups are labelled. Dots represent median ΔFrequency for individuals. Bar represents the median of the median ΔFrequency for each group. Representative plots from individuals showing changes in flagellar ear mechanical tuning frequency over 35 min (right) following Ringer (top) or 5 mM serotonin (bottom) injection. Injection occurred immediately after the first recording at zero min. Dots represent frequency values extracted from collected data. Solid lines in the right panels represent loess fits to these data points. **(D)** Median ΔFrequency data (top left) following 25 mM serotonin injection prior to a second injection of either Ringer (left), or 5 mM methiothepin (right) male injection groups. No significant differences between groups were identified *via* ART ANOVA. Dots represent median ΔFrequency for individuals and bar represents the median of the median ΔFrequency for each group. Minimum ΔFrequency (Min. ΔFrequency) data (top right) after a second injection of either Ringer (left), or 5 mM methiothepin (right) male injection groups following 25 mM serotonin injection. Significant differences identified *via* ART ANOVA between groups are labelled. Dots represent min ΔFrequency for individuals and bar represents the median of the min ΔFrequency for each group. Representative plots from individuals that show the time series of changes in flagellar ear mechanical tuning frequency (ΔFrequency) over the 40 min time course (bottom) of a primary injection of 25 mM serotonin followed by a secondary injection of either Ringer (left) or 5 mM methiothepin (right) injection. 25 mM serotonin injection occurred immediately after the first recording at zero min, and the second injection occurred immediately after the recording at 10 min. Dots represent frequency values extracted from collected data. Solid lines in the bottom panels represent loess fits to these data points. **(B–D)** ns, *p* > 0.05; *, *p* ≤ 0.05; **, *p* ≤ 0.01; ***, *p* ≤ 0.001.

Frequency matching between the mechanical tuning of the male flagellar vibration and the female flight sound facilitates male phonotaxis (Göpfert and Robert, 2000; Andrés et al., 2020). Manipulation of neurotransmitter systems *via* thoracic injection-mediated compound exposure allows for investigation of the potential involvement of certain neurotransmitters/receptors in determining mosquito hearing function. Furthermore, pharmacological saturation of these systems could theoretically provide a means for exploring the extent to which certain neurotransmitter/receptor combinations may be involved in shifting the mechanical frequency tuning of mosquito ears. Using established laser Doppler vibrometry based protocols, we tested changes in ear mechanical frequency tuning of both sexes over time both prior to and after compound injection (Figure 3A) (Su et al., 2018).

We first injected serotonin at different concentrations to examine its influence on the mechanical tuning frequency of male and female ears. As compared to the injection of a control Ringer solution, injection of 5 mM serotonin resulted in an increase in male ear mechanical tuning frequency. The median of the median ΔFrequency, that is the median across multiple individuals of the median frequency change (i.e., median ΔFrequency) in the 20 min following injection, was estimated at ∼75 Hz when as compared to Ringer controls (Figure 3B; Supplementary Figure S3A; Supplementary Table S3). The effect of 5 mM serotonin was almost immediate and resulted in a sustained shift in tuning frequency (Supplementary Figure S3A). Injection of 25 mM serotonin resulted in an increased median ΔFrequency compared to both Ringer (*p* < 0.001, ART ANOVA) and 5 mM serotonin injection (*p* < 0.01, ART ANOVA), with a median frequency shift of approximately 150 Hz as compared to Ringer controls (Figure 3B; Supplementary Table S3).

Individual time series data further reveals that the injection of higher concentration serotonin appeared to create a more prolonged shift in the male ear mechanical tuning frequency (Supplementary Figure S3A).

On the other hand, injection of 25 mM of alpha-methyltryptophan (AMTP) solution [a serotonin synthesis inhibitor, which should theoretically prevent novel serotonin synthesis (Sloley and Orikasa, 1988; Stevenson et al., 2000)] into males resulted in a significant decrease in frequency tuning of the ear (i.e., a negative median ΔFrequency) of approximately 65 Hz as compared to Ringer control (*p* < 0.001, ART ANOVA).

In females, injection of Ringer solution resulted in a median median ΔFrequency of −3.1 Hz. Injection of 5 mM serotonin resulted in a statistically significant change in frequency tuning as compared to the Ringer injection (median median ΔFrequency = 7.8 Hz, *p* < 0.05, ART ANOVA; Figure 3C; Supplementary Table S4). Serotonin modulation thus appears to have some influence on female ear mechanical tuning frequency (Supplementary Figure S3B).

Since we observed a significant increase in the male ear mechanical tuning frequency upon serotonin exposure, we next tested if we could reverse this effect by injecting a non-selective 5-HT receptor antagonist, methiothepin (Misane et al., 2000; Guseva et al., 2014). As a control, injection of Ringer solution following 25 mM serotonin injection led to a small decrease of around 40 Hz in frequency tuning in males, likely due to an associated reduction in serotonin concentration (shown as Min. ΔFrequency in Figure 3D top-right; Supplementary Figure S3C; Supplementary Table S5). Interestingly, injection of methiothepin following serotonin injection resulted in a significantly greater decrease (∼70 Hz of Min. ΔFrequency) in frequency tuning than Ringer injection (*p* < 0.05; ART ANOVA; Figure 3D top-right; Supplementary Figure S3C; Supplementary Table S5).

In summary, these results reveal that agonizing the serotonin receptors can lead to significant shifts of the male ear mechanical tuning frequency, which can be partially reversed *via* antagonist exposure. Furthermore, preventing serotonin synthesis *via* AMTP injection leads to the opposite effect on male ear frequency tuning compared to exposure to an agonist.

### Male hearing behaviors are altered following exposure to serotonin-synthesis inhibitor

Male attraction to the sound of flying, conspecific females is a clear, highly reproducible behavior (Andrés et al., 2020). Indeed, male attraction to female flight sounds is so strong that males can even be attracted to speakers playing pure tones of relevant frequencies; this frequency range is typically relatively narrow, with changes in pure tone frequency of >50 Hz leading to significant reductions in phonotactic response (Su et al., 2020). Male phonotaxis occurs predominantly within swarms, which form at specific times of the day and whose formation may be linked to serotonin expression levels (Anstey et al., 2009; Somers et al., 2022).

In comparison to males, female hearing behaviors remain almost entirely unknown, with no confirmed reports of female *Ae. aegypti* phonotaxis. Furthermore, although serotonin injection did result in a statistically significant shift in female ear mechanical frequency tuning, the median effect size of this modulation was less than 10 Hz (as compared to the estimated 80 Hz shift seen for males). We thus decided to focus on the effect of serotonin modulation on male hearing behaviors, given their well-documented, reproducible nature and the far larger effect of serotonin on male hearing function.

**FIGURE 4:**
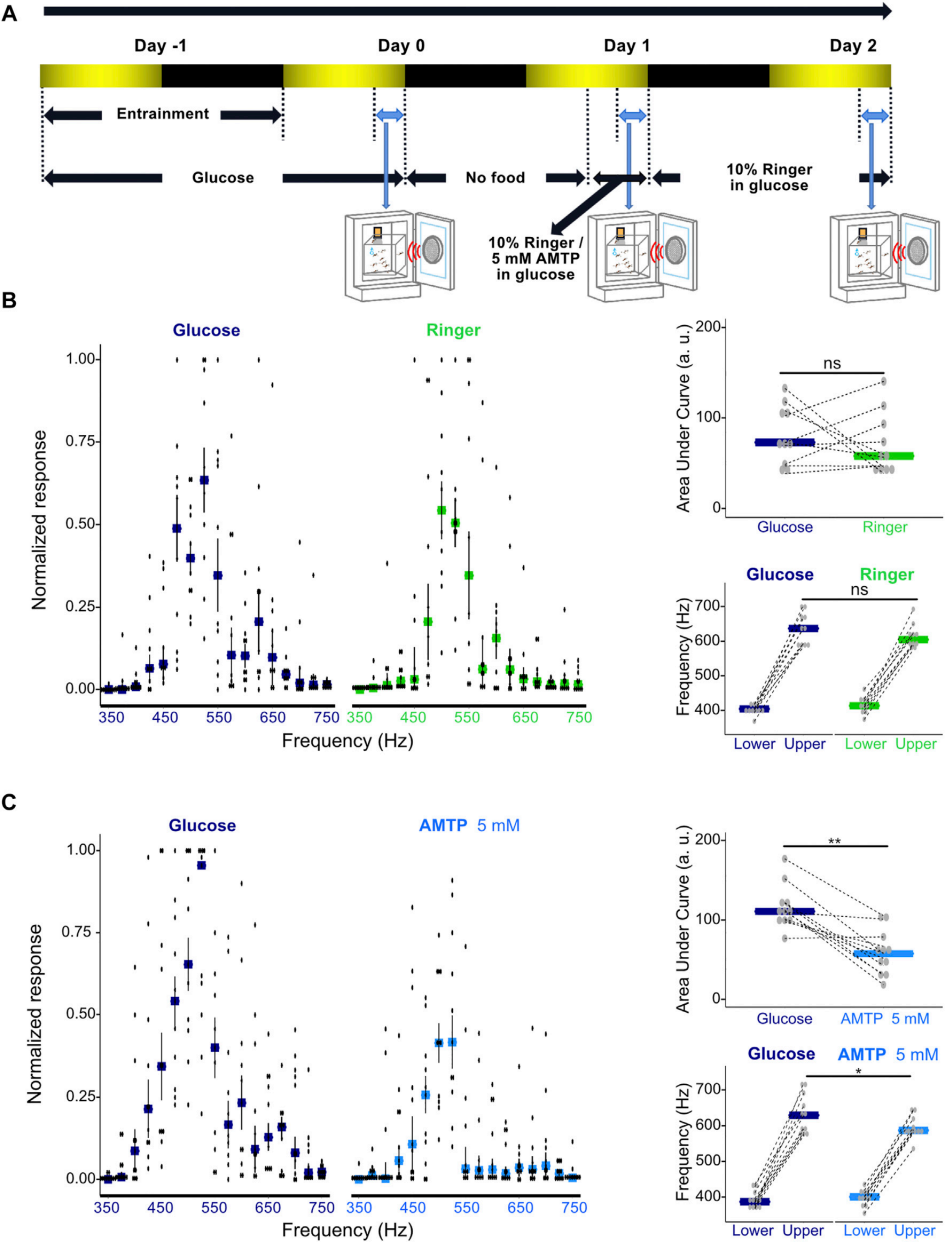
Modulation of serotonin synthesis significantly alters male phonotactic behavior. **(A)** Diagram of phonotaxis testing paradigm. Phonotactic behavior was tested at dusk (ZT 12, indicated by blue bidirectional arrows). Baseline phonotactic behavior was recorded at Day 0 as a glucose-treated group. Phonotactic behavior after compound feeding was recorded at Day 1 as a Ringer or alpha-methyltryptophan (AMTP)-treated group. **(B)** Normalized phonotactic responses (left) for glucose (left, measured at Day 0) and Ringer (right, measured at Day 1) fed male mosquitoes. Bars represent median values for each frequency, error bars represent SEM, and dots represent values from individual repeats. Changes in the area under the curve (AUC) of male phonotactic profile (top right) (AUC) between glucose (Day 0) and Ringer (Day 1) exposures. No significant differences were identified between groups using Wilcoxon signed rank tests. Bars represent the median values for each group and dots represent the values for individual repeats. Dependent-data points collected from the same repeat are connected *via* dotted lines. Changes in the frequency range (bottom right) to which males respond to between glucose (Day 0) and Ringer (Day 1) exposures. Lower and upper frequency boundaries are shown. No significant differences were identified between groups using Wilcoxon signed rank tests. Bars represent the median values for each group and dots represent the values for individual repeats. Dependent-data points collected from the same repeat are connected *via* dotted lines. **(C)** Normalized phonotactic responses (left) for glucose (left, measured on Day 0) and 5 mM AMTP (right, measured on Day 1) fed male mosquitoes. Bars represent median values for each frequency, error bars represent SEM, and dots represent values from individual repeats. Changes in AUC of male phonotactic profile (top right) between glucose (Day 0) and 5 mM AMTP (Day 1) exposures. Significant differences identified between groups using Wilcoxon signed rank tests are labelled. Bars represent the median values for each group and dots represent the values for individual repeats. Dependent-data points collected from the same repeat are connected *via* dotted lines. Changes in the frequency range to which males respond to between glucose (Day 0) and 5 mM AMTP (Day 1) exposures. Lower and upper frequency boundaries are shown. Significant differences identified between groups using Wilcoxon signed rank tests are labelled. Bars represent the median values for each group and dots represent the values for individual repeats. Dependent-data points collected from the same repeat are connected *via* dotted lines. ns, *p* > 0.05; *, *p* ≤ 0.05; **, *p* ≤ 0.01.

To investigate the potential effects of serotonin modulation on both the extent of phonotactic attraction and the range of frequencies males were attracted to, we tested the responses of groups of 30 *Ae. aegypti* males to frequencies of sound between 350 and 750 Hz in 25-Hz increments presented in a randomized order. Males were first tested when exposed only to glucose food as a baseline, and then starved for 21 h before being provided with doped food containing either Ringer solution or 5 mM AMTP, a serotonin synthesis inhibitor (Sloley and Orikasa, 1988; Stevenson et al., 2000; Dierick and Greenspan, 2007; Anstey et al., 2009). Males were then tested for the second time 3 h after compound exposure (Figure 4A). Global reduction of serotonin levels in AMTP-exposed males was confirmed *via* ELISA assay, with males exposed to 5 mM or 25 mM AMTP showing a strong trend of decreased serotonin concentration as compared to Ringer-exposed control males (Supplementary Figure S4).

Feeding male *Ae. aegypti* a Ringer control solution did not lead to a significant change in their overall response level as determined by AUC analysis (*p* = 0.1563, Wilcoxon signed rank test; Figure 4B; Supplementary Table S6) or the range of frequencies males responded to (*p* > 0.05, Wilcoxon signed rank test; Figure 4B; Supplementary Table S7).

Chronic exposure to 5 mM AMTP over 3 h however both reduced the magnitude of responses to sound (*p* < 0.05, Wilcoxon signed rank test; Figure 4C; Supplementary Table S6) and the range of frequencies to which males responded to (*p* < 0.05, Wilcoxon signed rank test; Figure 4C; Supplementary Table S7). Providing males with Ringer-doped food after AMTP exposure allowed for apparent partial recovery of their phonotactic ability and restored responses to a wider range of frequencies (Supplementary Figure S5A; Supplementary Table S8).

These results demonstrate that global pharmacological manipulation of serotonin levels in *Ae. aegypti* males can alter both their overall phonotactic response level and the frequency range to which they respond.

## Discussion

The emergence and rapid development of insecticide-resistance across multiple species of mosquitoes has jeopardized current mosquito control programs (Liu, 2015). New tools with novel targets are therefore of desperate need to control the mosquito populations (Killeen et al., 2017). Given the importance of phonotaxis in mosquito mating, targeting male hearing is one promising option (Andrés et al., 2020; Staunton et al., 2021) Here, we explored the possibility of manipulating the male *Ae. aegypti* serotonergic system as a means of inhibiting male hearing function and its relevant behaviors.

### Serotonin and presynaptic sites overlap strongly in female, but not male, JOs

Male and female JOs differ significantly both anatomically and functionally. The differences we found in terms of JO size between sexes agree with previous studies (Boo and Richards, 1975a; Boo and Richards, 1975b). Furthermore, sexual dimorphism in the distribution patterns of the presynaptic marker 3C11 in the JOs are also in accordance with previous publications: male *Ae. aegypti* show dense synaptic punctae in the gap between cilia and somata, while in females, signals were mostly restricted to the somata (Su et al., 2018).

Interestingly, the location of serotonin expression in both male and female JOs appears to be less- or non-sexually dimorphic: serotonin is predominantly found in the somata of JO neurons in both sexes. Signal co-localizations between serotonin and 3C11 (anti-SYNORF1) were found in both the somata of male and female JOs, albeit at a relatively reduced level in the males (Supplementary Figure S1B). These presynaptic sites may therefore be serotonergic and may be involved in the releasing of serotonin into the JO. Furthermore, the abundant non-serotonin labelled synaptic punctae found in the gap between the cilia and somata may have other neurochemical profiles, such as being octopaminergic (Andrés et al., 2016).

Given the clear sexual dimorphisms in male and female JOs, we wondered if the fundamental projection pattern of JO neurons to the brain remains conserved between the sexes. Projection patterns of the JO and flagellar neurons, mapped *via* anterograde Dextran injection, seemed similar in both sexes; flagellar neurons appeared to project to the AL region, whilst JO neurons send their axons to the AMMC. The latter result contradicts a previous report indicating that JO neurons project to a non-glomerular region in the AL, previously denoted as JO-C (Ignell et al., 2005). Our images instead strongly indicate that JO neurons innervate the AMMC, a neuropil diagonally lateral to the AL (Nyhof and McIver, 1989; Childress and McIver, 2011). Previous reports investigating the structure of AL in mosquitoes also failed to observe the non-glomerular JO-C; instead, the putative JO-C site was found to be comprised of distinct, individual glomeruli (Riabinina et al., 2016; Shankar and McMeniman, 2020).

### Relative overexpression of 5-HT_7_ family receptors in the JO suggests a potential role in determining hearing function

Building on this anatomical data, we identified gene expression of six serotonin receptors in male and female *Ae. aegypti* JOs. In both sexes, only the two members of the 5-HT_7_ subfamily were found to have a significantly higher expression in the JOs relative to the head. This receptor family, which has been linked to various important behaviors including mating and sleeping, is of interest for future studies (Becnel et al., 2011; Liu et al., 2019), particularly as both knockdown and pharmacological antagonization of 5-HT_7_ receptors in *D. melanogaster* significantly reduced courtship (Becnel et al., 2011).

The previous identification of two 5-HT_7_ family members in mosquitoes, in contrast to the single 5-HT_7_ receptor found in *Drosophila*, also raises important questions regarding potential functional differences between these two subfamily receptors. A lack of receptor-specific antibodies hinders the localization of these receptors within the JO. Generating knockout mutants for individual receptors *via* CRISPR-Cas9-mediated mutagenesis will be necessary to help identify the receptor(s) influencing mosquito hearing function.

Previous work in *D. melanogaster* demonstrated that JO neurons are mostly cholinergic (Ishikawa et al., 2017). Here, we identified local expression of two serotonergic neuronal markers, *TPH* and *SERT*, within the JOs of both sexes (Supplementary Figures S2B,C). Along with our serotonin IHC staining (Figure 1D), this raises the possibility that some JO neurons in *Ae. aegypti* could be serotonergic. Taken together, our results suggest that serotonin could be involved in both afferent and efferent auditory signaling pathways in *Ae. aegypti*.

### The distinct natures and functions of different serotonin receptor families may influence male ear mechanical tuning

To examine the functional relevance of serotonin and serotonin receptor expression in the ears of *Ae. aegypti*, we performed multiple series of individual or dual serotonin-related compound injections and measured the mechanical tuning frequency of the ears. Injection of 5 mM serotonin significantly increased the tuning frequency of male ears by around ∼75 Hz, with this effect wearing off in approximately 1 h (data not shown). Injection of 25 mM serotonin showed an apparent extension based on the effect time plots (Supplementary Figure S3A), suggesting that prolonged activation of the serotonin signaling pathway is essential for maintaining and stabilizing the increased mechanical tuning frequency. Furthermore, injection of 25 mM 5-HTP (a precursor to 5-HT) also led to a significant increase in the male ear mechanical tuning frequency of approximately 135 Hz (Supplementary Figure S6; Supplementary Table S3).

Since serotonin exposure increases the mechanical tuning frequency of male ears, we hypothesized that injection of a serotonin synthesis inhibitor, AMTP, would do the exact opposite (Sloley and Orikasa, 1988). Indeed, injection of 25 mM AMTP significantly reduced male ear mechanical tuning frequency within the 30 min duration of recordings. Since AMTP acts to prevent novel serotonin synthesis (Figure 3A), this change in the ear mechanical tuning frequency may be the indirect result of a reduced serotonin level. Constant, relatively low levels of serotonin in the male JO may thus be required to maintain the male ear mechanical tuning at a baseline frequency at all times of the day, with specific, timely release of greater quantities of serotonin leading to a significant increase in the tuning frequency.

Female *Ae. aegypti* subjected to similar testing showed a small but significant change in their ear mechanical tuning as compared to Ringer injected controls (∼10 Hz). As female human-biting mosquitoes have yet to be reported to display any apparent phonotactic behavior, the behavioral relevance of this modulation is unclear (Su et al., 2018; Somers et al., 2022). Female mosquito hearing in general remains poorly understood and will require further detailed analyses focusing not only on their peripheral function but also the central processing of sound information.

In both insects and mammals, *via* association with unique G proteins, receptors from different 5-HT families differentially modulate the dynamics of the neural network by activating distinct intracellular signaling pathways (Millan et al., 2008). Upon activation by serotonin, members of the 5-HT_1_ family have been reported to exhibit an inhibitory role in target neurons, which is achieved by inhibiting cyclic AMP *via* the Gαi/o protein (Blenau and Baumann, 2001). Furthermore, 5-HT_1_ receptors have been found to be expressed presynaptically and possess potential autoreception function in *Drosophila* (Witz et al., 1990; Saudou et al., 1992; Blenau and Baumann, 2001; Yuan et al., 2005). In other words, the increase in binding of serotonin to members of the 5-HT_1_ family should lead to a concomitant reduction in the release of serotonin from these presynaptic terminals due to the inhibitory nature of the receptors. Two putative 5-HT_1_ family receptors of *Ae. aegypti*, AAEL008360 (5-HT_1A_) and AAEL017272 (5-HT_1B_), may share this function, as these receptor proteins are highly homologous to their *D. melanogaster* counterparts (Supplementary Figure S2).

On the other hand, *Ae. aegypti* 5-HT_2_ (AAEL019805 and AAEL019804) and 5-HT_7_ (AAEL027242 and AAEL025125) subfamily members respectively exhibited homology to *D. melanogaster* 5-HT_2_ and 5-HT_7_ family receptors. Based on previous *D. melanogaster* studies, these two receptor families may functionally increase intracellular calcium and cAMP levels by exerting their effect *via* the Gαq/11 and Gαs proteins, respectively (Figure 3A) (Witz et al., 1990; Saudou et al., 1992; Blenau and Baumann, 2001; Yuan et al., 2005; Becnel et al., 2011; Sampson et al., 2020).

Injection of methiothepin, a non-selective serotonin receptor antagonist, partially reverted males toward the prior tuning state, possibly *via* competing for 5-HT_2_ and 5-HT_7_ receptors with serotonin (Figures 3A,D) (Guseva et al., 2014). If methiothepin binds to members of the 5-HT_1_ subfamily, an increase in serotonin release coupled with an associated upward shift in the male ear mechanical tuning should be expected. However, our results do not favor such a hypothesis; we observed no increase in male ear tuning frequency following antagonist injection, and 5-HT_1_ family members did not appear to be significantly highly expressed in the male JO compared to the head tissue. This does not however rule out the possibility that some serotonin was released following the binding of methiothepin to 5-HT_1_ receptors, preventing the male ear mechanical tuning frequency from returning to a baseline state.

The significantly higher relative expression of 5-HT_7_ subfamily members in the JO compared to the head in both sexes, as opposed to other serotonin receptors, suggests that they may be involved in the effect of serotonin on hearing function. Functional studies of these receptors *via* mutagenesis, coupled with antibody localization of these receptors, may shed light on the molecular mechanism(s) underlying the action of serotonin on male peripheral hearing function of males. Members of the 5-HT_7_ subfamily therefore represent druggable targets for future pharmacological and mutagenesis studies. (Fuchs et al., 2014; Audsley and Down, 2015).

### Chronic, global disruption of serotonin synthesis can significantly alter core male mosquito behaviors

Previous papers have reported that chronic exposure of cockroaches or flies to AMTP, a serotonin synthesis inhibitor, led to a significant reduction in serotonin levels in the brain; 4-day exposure of flies to 5 mM AMTP resulted in a 20% reduction in the serotonin level in fly heads for example (Stevenson et al., 2000; Dierick and Greenspan, 2007). We confirmed *via* ELISA that estimated baseline levels of serotonin in males feeding from ringer-doped food appeared within the physiological range of 5-HT concentrations previously observed in *Ae. aegypti* (Ling and Raikhel, 2018). This lends credence to the apparent reduction in serotonin levels seen for males exposed to different concentrations of AMTP as compared to controls during dusk (when swarming is likely to occur) (Supplementary Figure S4).

After a 3 h oral administration of AMTP prior to dusk, we observed that *Ae. aegypti* males showed a clear reduction in their overall phonotactic response level (Figure 4C; individual phonotactic profiles included in Supplementary Figure S7). This overall reduction in phonotactic response level (as quantified by changes in AUC) could be the direct consequence of reduced flight and locomotor activity resulting from global depletion of serotonin levels (Stevenson et al., 2000; Dasari et al., 2007). Besides an overall reduction in the overall phonotactic response level in serotonin-depleted males, we also found that these males exhibited responses to a narrower range of tones following AMTP administration (Figure 4C). Given that injection of 25 mM AMTP resulted in a significant decrease in male ear mechanical tuning frequency, this likely indicates that chronic prevention of serotonin synthesis can alter the phonotactic profiles of males *via* altering their hearing function.

We therefore hypothesize that the timely synthesis and release of novel serotonin during the aggregation (swarming) period (Anstey et al., 2009) may be correlated with a functional increase in the flagellar ear mechanical tuning frequency of males that facilitate their phonotactic response to a wider range of tones. To lend support to such a hypothesis, our preliminary tests on the phonotactic behavior of control males during non-swarming periods, when serotonin expression is presumably low, indicated that whilst males still show some response to sound, the magnitude of this response is considerably reduced and the response range is also narrower (Supplementary Figure S5B; individual repeat profiles included in Supplementary Figure S7).

Taken together, our work provides the first evidence that interfering with male mosquito neurotransmitter function can alter significant hearing behaviors. Further improvements to our understanding of the underlying bases of serotonin’s effect on mosquito hearing function, as well as improving future phonotaxis assays to enable high-throughput testing, is now necessary to help translate these findings for use in field settings.

## Data Availability

All raw data and analysis scripts used in the analyses will be made available by the authors upon request.

## Supplementary material

Supplementary Figure S1
**(A)** Horizontal sections of male (left) and female (right) ears (Johnston's organ (JO) and flagellum). anti-horseradish peroxidase (HRP) staining (magenta) allows for visualizing neurons, anti-serotonin staining (green) shows location of serotonin. Yellow frames in the left panels for each sex indicate the positions for the right panels. Right panels show individual channels for HRP and serotonin. **(B)** Horizontal sections of male (left) and female (right) JOs showing overlap between the presynaptic marker, anti-SYNORF1 (3C11) and serotonin. HRP staining (magenta) allows for visualizing neurons, 3C11 staining (blue) shows synaptic punctae and serotonin staining (green) shows location of serotonin. Yellow frames in the left panels for each sex indicate the positions for the right panels. Right panels show only 3C11 and serotonin channels. **(A,B)** Scale bar = 50 μm; A, anterior; M, medial.

Supplementary Figure S2
**(A)** Phylogenetic tree of *Drosophila melanogaster* (*D. melanogaster*), *Anopheles gambiae* (*An. gambiae*) and *Aedes aegypti* (*Ae. aegypti*) serotonin receptors. The *Ae. aegypti* *norepinephrine transporter* (AAEL005581) was selected as an outgroup gene. AA: *Ae. aegypti*; AG: *An. gambiae*; D: *Drosophila*. **(B)** RT-PCR showing the expression of *tryptophan hydroxylase* (*TPH*) in the indicated sexes and samples, male (top) and female (bottom) bodies, heads and Johnston's organ (JOs). +: positive control, ribosomal protein s7 (*rps7*), TPH and −: negative control, RNA (samples not reverse transcribed). Estimated sizes are 501 bp for *rps7* and 392 bp for *TPH*. Red arrow indicates the position of *TPH* signals. **(C)** RT-PCR showing the expression of *serotonin transporter* (*SERT*) in the indicated sexes and samples, male (top) and female (bottom) bodies, heads and Johnston's organ (JOs). −: negative control, RNA (samples not reverse transcribed), *SERT* and +: *rps7* Estimated size for *SERT* is 347 bp. Red arrow indicates the position of *SERT* signals..

Supplementary Figure S3
**(A)** Individual time series for changes in male flagellar ear mechanical frequency tuning following Ringer (top), 5 mM serotonin (top middle), 25 mM serotonin (bottom middle), and 25 mM alpha-methyltryptophan (AMTP) (bottom) injections. Dots represent frequency values extracted from collected data. Solid lines represent loess fits to these data points. **(B)** Individual time series for changes in female flagellar ear mechanical frequency tuning following Ringer (top) and 5 mM serotonin (bottom) injections. Dots represent frequency values extracted from collected data. Solid lines represent loess fits to these data points. **(C)** Individual time series for changes in male flagellar ear mechanical frequency tuning following 25 mM serotonin and Ringer (top), 25 mM serotonin and 5 mM methiothepin (middle), and 25 mM AMTP and 5 mM serotonin (bottom) injections. Dots represent frequency values extracted from collected data. Solid lines represent loess fits to these data points.

Supplementary Figure S4
**(A)** Boxplot of absolute serotonin concentration (in ng/ml) for groups of male mosquitoes which consumed 10% glucose food doped with either Ringer solution (left), 5 mM AMTP (middle) or 25 mM AMTP (right). Dots represent data for individual repeats. Bar represents the median value for each group.

Supplementary Figure S5
**(A)** Normalized phonotactic response (top) at dusk (ZT 12) for glucose (left), 5 mM alpha-methyltryptophan (AMTP) (middle) and Ringer (right) fed male mosquitoes. Bars represent median values for each frequency, error bars represent SEM, and small dots represent individual repeats. Changes in the area under the curve (AUC) of male phonotactic profile (bottom left) when males were provided with glucose, 5 mM AMTP-doped food or Ringer-doped food. Changes in the frequency range to which males showed response to (bottom right) when provided with glucose, 5 mM AMTP-doped or Ringer-doped food. Lower and upper frequency boundaries are shown. Bars represent median values, dots represent individual repeats and data collected from a single repeat are connected with dotted lines. **(B)** Normalized phonotactic response of males (left) tested at dawn (ZT 0) (left) and dusk (ZT 12) (right). Bars represent median values for each frequency, error bars represent SEM, and dots represent values from individual repeats. Individual male phonotactic profiles collected at dawn (ZT 0) and dusk (ZT 12) (top right and bottom right).

Supplementary Figure S6
**(A)** Median ΔFrequency data for Ringer and 25 mM 5-hydroxytryptophan (5-HTP) male injection groups. Significant differences identified *via* ART ANOVA between groups are labelled. Dots represent median ΔFrequency for individuals. Injection occurred immediately after the first recording at zero min. Bar represents the median of the median ΔFrequency for each group. ***, P ≤ 0.001. **(B)** Individual time series for changes in male flagellar ear mechanical frequency tuning following 25 mM 5-HTP injection. Dots represent frequency values extracted from collected data. Solid lines represent loess fits to these data points.

Supplementary Figure S7
**(A)** Individual phonotactic profiles of males that were first fed with glucose, followed by exposure to Ringer-doped food. **(B)** Individual phonotactic profiles of males that were first fed with glucose, followed by exposure to 5 mM alpha-methyltryptophan (AMTP)-doped food. **(C)** Individual phonotactic profiles of males that were first fed with glucose, followed by AMTP-doped food exposure and a subsequent exposure to Ringer-doped food.

## Abbreviations

5-HT, 5-hydroxytryptamine; 5-HTP, 5-hydroxytryptophan; *Ae. aegypti*, *Aedes aegypti*; AMTP, alpha-methyltryptophan; *An. gambiae*, *Anopheles gambiae*; AL, antennal lobe; AMMC, Antennal mechanosensory motor center; *D. melanogaster*, *Drosophila melanogaster*; HRP, horseradish peroxidase; IHC, immunohistochemistry; JO, Johnston's organ; NGS, normal goat serum; PBS, Phosphate-buffered saline; PBT, Triton X-100; PFA, paraformaldehyde; *rps7*, *ribosomal protein s7*; SERT, serotonin transporter; TPH, tryptophan hydroxylase; WBF, wing beat frequency; ZT, Zeitgeber.
