# Supplementary figures and images for: Serotonin modulation in the male Aedes aegypti ear influences hearing
Source: Front Physiol. 2022 Aug 29;13:931567. doi: 10.3389/fphys.2022.931567 (PMC9465180; doi:10.3389/fphys.2022.931567)

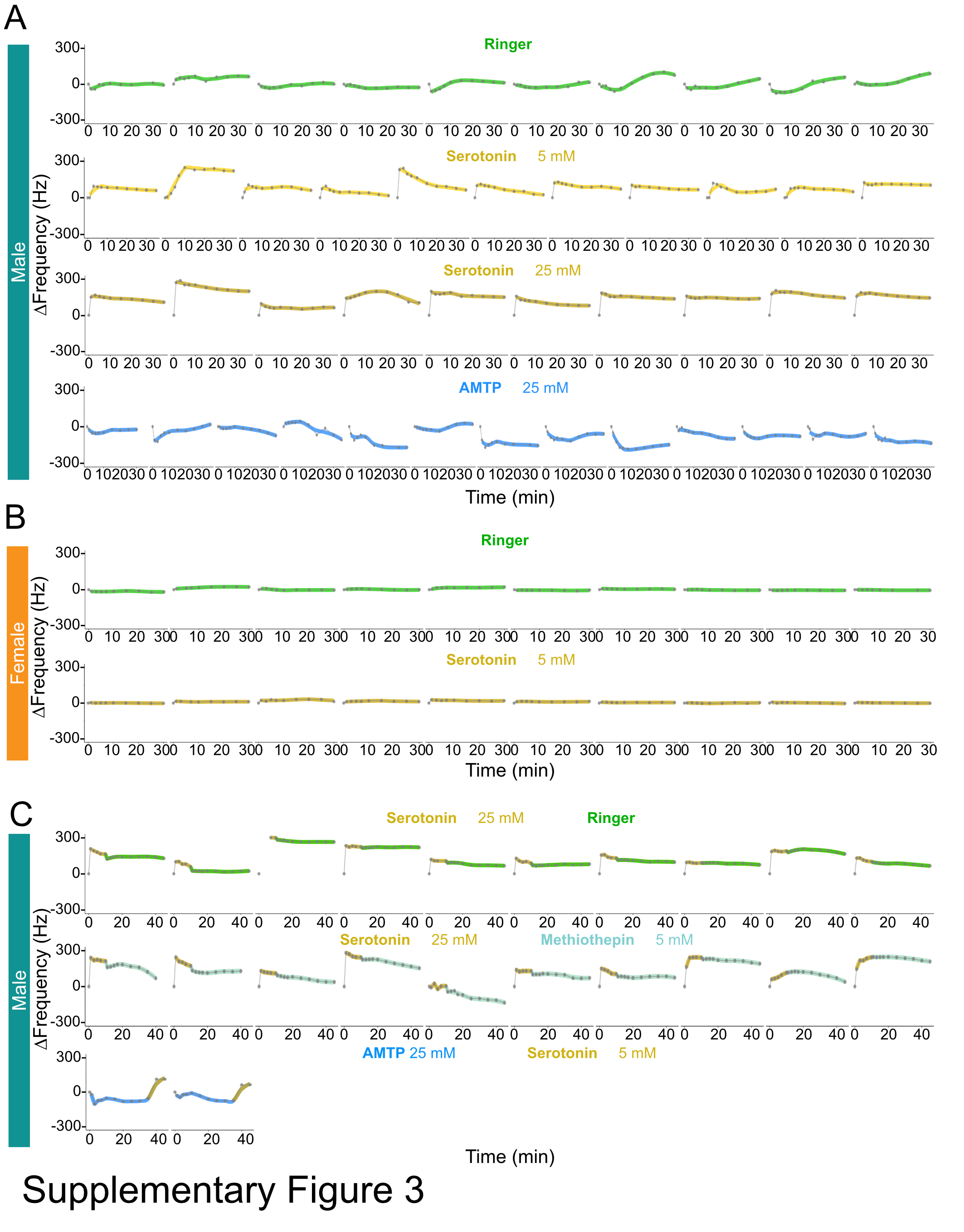

Supplement: Supplementary file 1 [file Image3.TIFF]

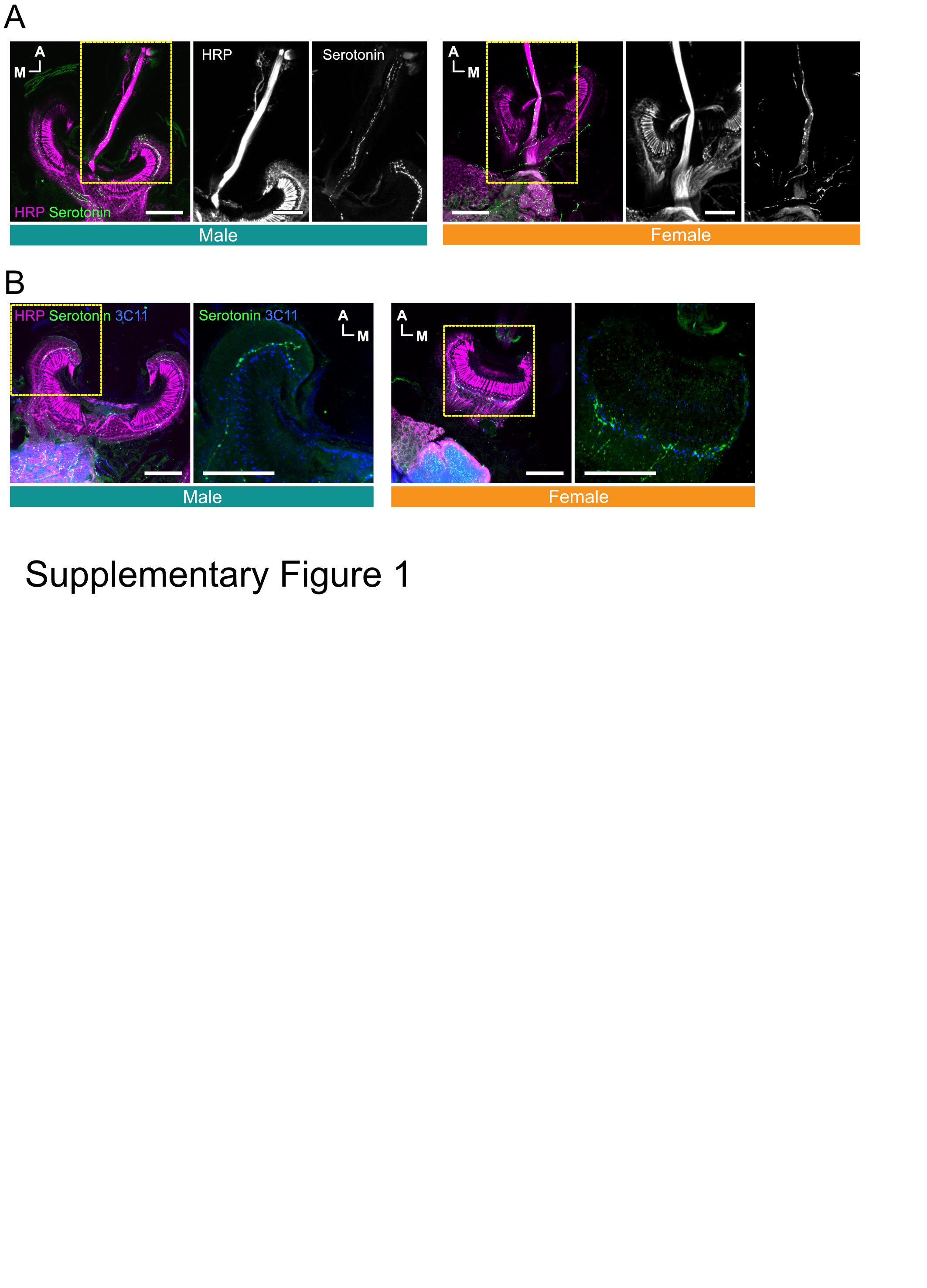

Supplement: Supplementary file 2 [file Image1.TIFF]

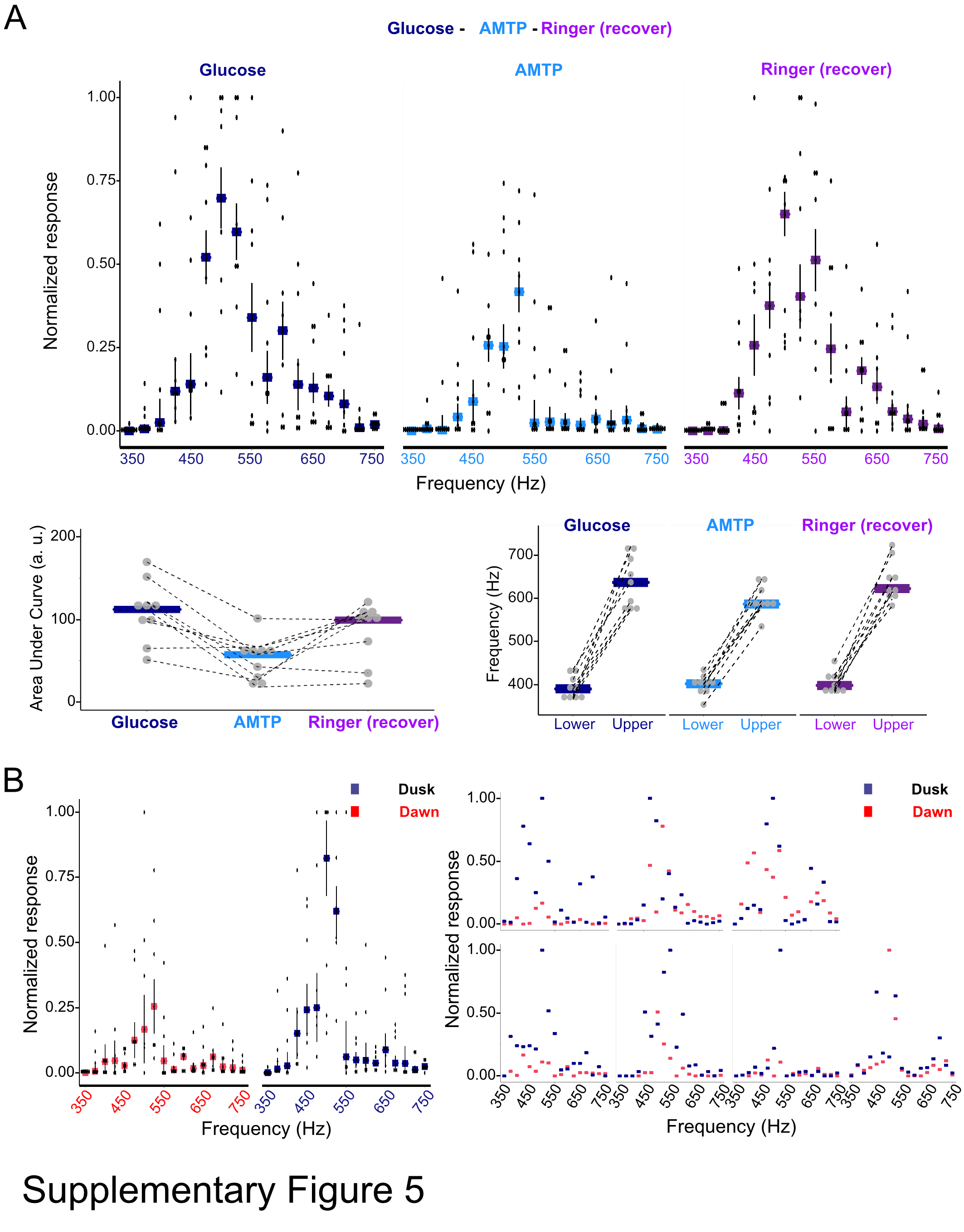

Supplement: Supplementary file 3 [file Image5.TIFF]

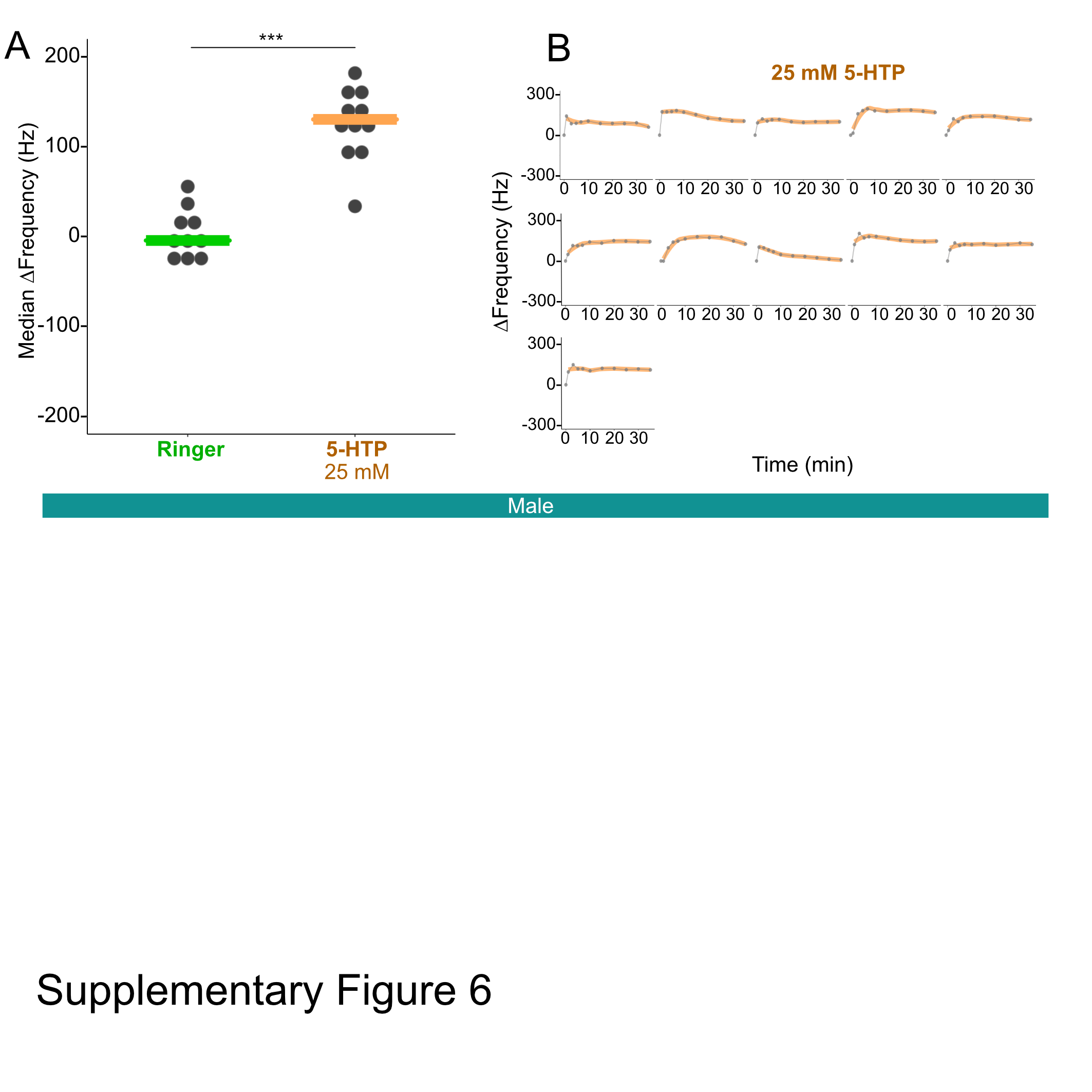

Supplement: Supplementary file 4 [file Image6.tiff]

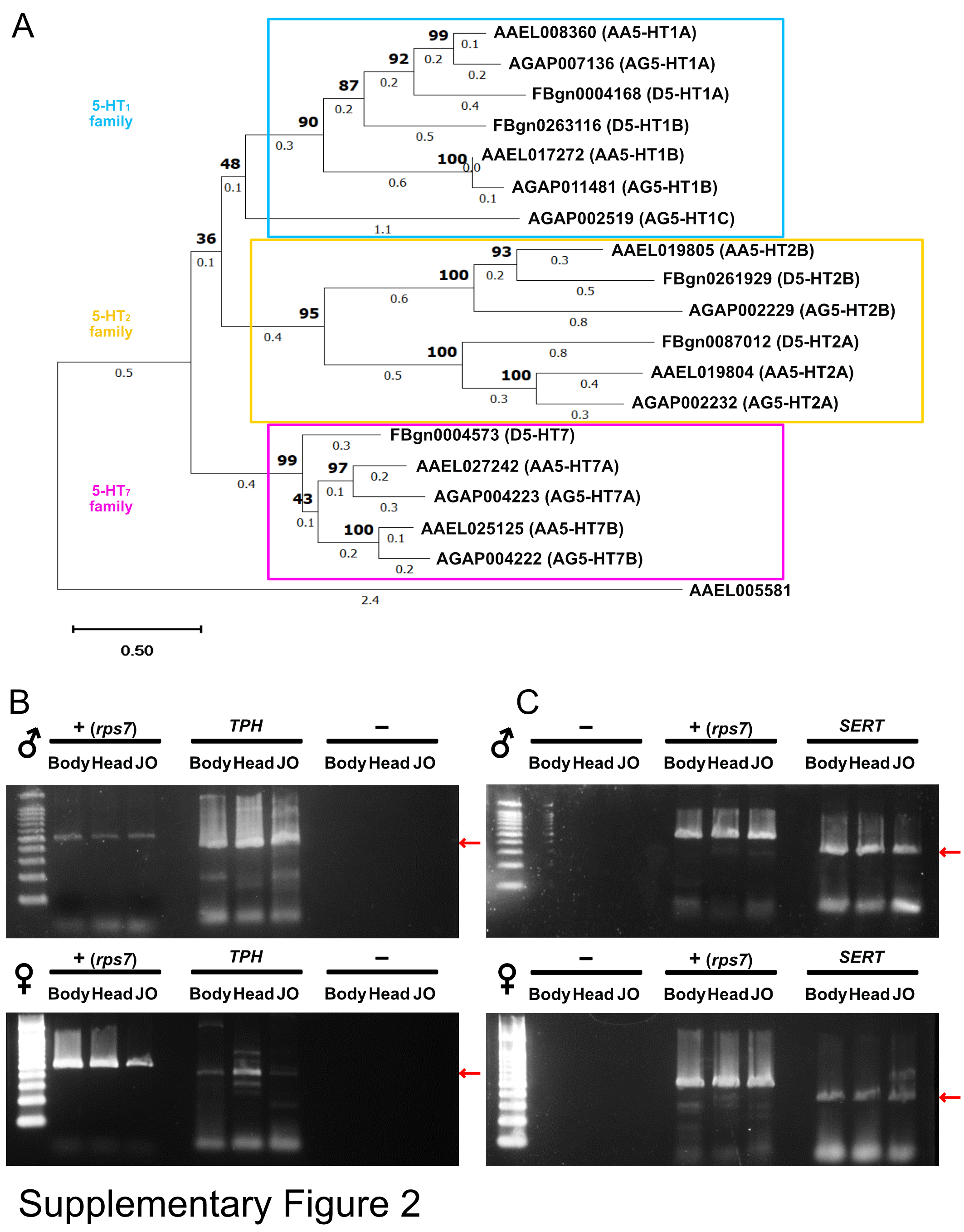

Supplement: Supplementary file 5 [file Image2.TIFF]

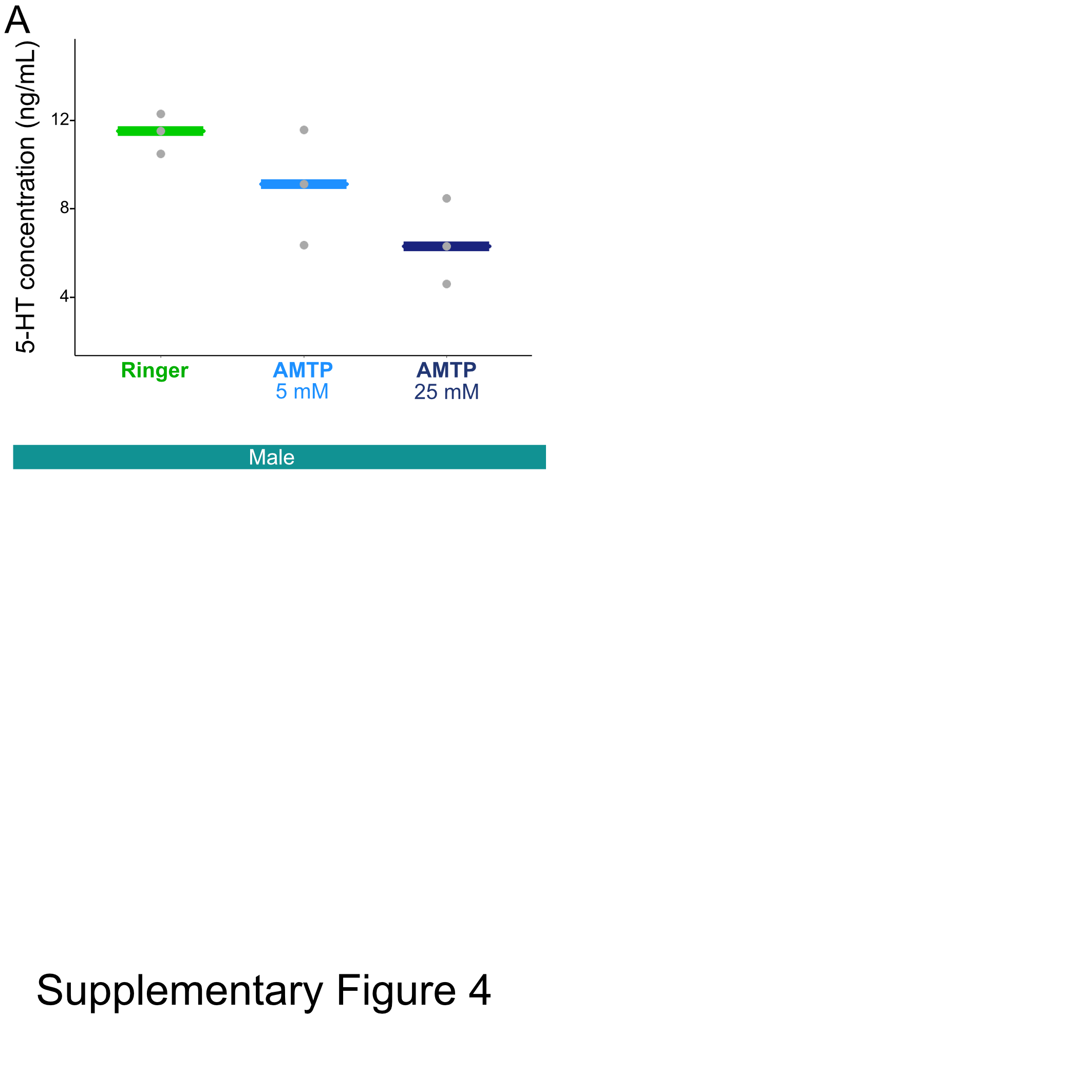

Supplement: Supplementary file 6 [file Image4.TIFF]

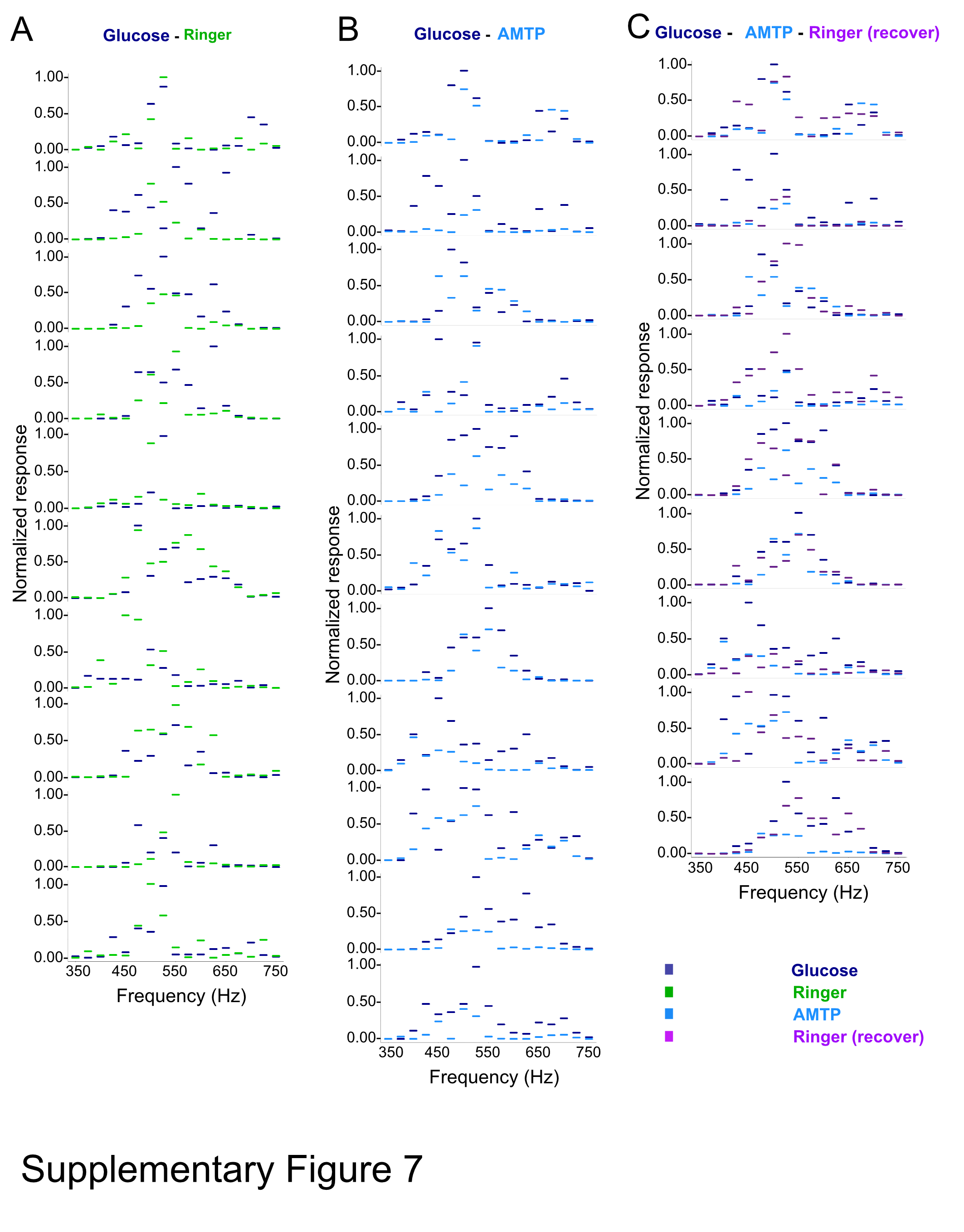

Supplement: Supplementary file 8 [file Image7.tiff]
