# Supplementary material for: Serotonin modulation in the male Aedes aegypti ear influences hearing
Source: Front Physiol. 2022 Aug 29;13:931567. doi: 10.3389/fphys.2022.931567 (PMC9465180; doi:10.3389/fphys.2022.931567)
Supplement: Supplementary file 7 [file DataSheet1.docx]

**Supplemental Table 1: Statistical comparisons for female qPCR data**

Statistical comparisons (calculated using Repeat ANOVAs) for each primer for female *Ae. aegypti* tissues. Stars indicate significance level (* p<0.05, ** p<0.01, *** p<0.001).

| **5-HT_1A_** | |  |  |  |  |  |
| --- | --- | --- | --- | --- | --- | --- |
| **Tissue 1** | **Tissue 2** | **Sample size** | **Statistic** | **df** | **p value** | **Adj p value** |
| Body | Head | 7 | 11 | 6 | 0.00003 | 0.00010*** |
| Body | JO | 7 | 8.2 | 6 | 0.00018 | 0.00053*** |
| Head | JO | 7 | -1.19 | 6 | 0.278 | 0.834 |
| **5-HT_1B_** | |  |  |  |  |  |
| **Tissue 1** | **Tissue 2** | **Sample size** | **Statistic** | **df** | **p value** | **Adj p value** |
| Body | Head | 7 | 11.6 | 6 | 0.00002 | 0.00007*** |
| Body | JO | 7 | 8.78 | 6 | 0.00012 | 0.00036*** |
| Head | JO | 7 | -2.02 | 6 | 0.089 | 0.268 |
| **5-HT_2A_** | |  |  |  |  |  |
| **Tissue 1** | **Tissue 2** | **Sample size** | **Statistic** | **df** | **p value** | **Adj p value** |
| Body | Head | 7 | 4.81 | 6 | 0.003 | 0.009** |
| Body | JO | 7 | 3.92 | 6 | 0.008 | 0.023* |
| Head | JO | 7 | 0.302 | 6 | 0.773 | 0.773 |
| **5-HT_2B_** | |  |  |  |  |  |
| **Tissue 1** | **Tissue 2** | **Sample size** | **Statistic** | **df** | **p value** | **Adj p value** |
| Body | Head | 7 | 5.68 | 6 | 0.001 | 0.004** |
| Body | JO | 7 | 4.55 | 6 | 0.004 | 0.012* |
| Head | JO | 7 | 1.02 | 6 | 0.348 | 1 |
| **5-HT_7A_** | |  |  |  |  |  |
| **Tissue 1** | **Tissue 2** | **Sample size** | **Statistic** | **df** | **p value** | **Adj p value** |
| Body | Head | 7 | 8.71 | 6 | 0.00013 | 0.00038*** |
| Body | JO | 7 | 9.42 | 6 | 0.00008 | 0.00025*** |
| Head | JO | 7 | 7.57 | 6 | 0.00028 | 0.00083*** |
| **5-HT_7B_** | |  |  |  |  |  |
| **Tissue 1** | **Tissue 2** | **Sample size** | **Statistic** | **df** | **p value** | **Adj p value** |
| Body | Head | 7 | 5.95 | 6 | 0.001 | 0.003** |
| Body | JO | 7 | 6.62 | 6 | 0.00057 | 0.002** |
| Head | JO | 7 | 7.21 | 6 | 0.00036 | 0.001** |

**Supplemental Table 2: Statistical comparisons for male qPCR data**

Statistical comparisons (calculated using Repeat ANOVAs) for each primer for male *Ae. aegypti* tissues. Stars indicate significance level (* p<0.05, ** p<0.01, *** p<0.001).

| **5-HT_1A_** | |  |  |  |  |  |  |
| --- | --- | --- | --- | --- | --- | --- | --- |
| **Tissue 1** | **Tissue 2** | **Sample size** | **Statistic** | **df** | **p value** | **Adj p value** |  |
| Body | Head | 7 | 5.58 | 6 | 0.001 | 0.004** |  |
| Body | JO | 7 | 2.64 | 6 | 0.039 | 0.116 |  |
| Head | JO | 7 | -6.27 | 6 | 0.000766 | 0.002** |  |
| **5-HT_1B_** |  |  |  |  |  |  |  |
| **Tissue 1** | **Tissue 2** | **Sample size** | **Statistic** | **df** | **p value** | **Adj p value** |  |
| Body | Head | 7 | 6.94 | 6 | 0.000445 | 0.001** |  |
| Body | JO | 7 | 9.93 | 6 | 0.0000601 | 0.00018*** | |
| Head | JO | 7 | 1.99 | 6 | 0.094 | 0.283 |  |
| **5-HT_2A_** |  |  |  |  |  |  |  |
| **Tissue 1** | **Tissue 2** | **Sample size** | **Statistic** | **df** | **p value** | **Adj p value** |  |
| Body | Head | 7 | 3.38 | 6 | 0.015 | 0.045* |  |
| Body | JO | 7 | 2.21 | 6 | 0.07 | 0.209 |  |
| Head | JO | 7 | -0.29 | 6 | 0.782 | 1 |  |
| **5-HT_2B_** |  |  |  |  |  |  |  |
| **Tissue 1** | **Tissue 2** | **Sample size** | **Statistic** | **df** | **p value** | **Adj p value** |  |
| Body | Head | 7 | 3.03 | 6 | 0.023 | 0.069 |  |
| Body | JO | 7 | 1.55 | 6 | 0.172 | 0.516 |  |
| Head | JO | 7 | -2.73 | 6 | 0.034 | 0.103 |  |
| **5-HT_7A_** | |  |  |  |  |  |  |
| **Tissue 1** | **Tissue 2** | **Sample size** | **Statistic** | **df** | **p value** | **Adj p value** |  |
| Body | Head | 7 | 2.51 | 6 | 0.046 | 0.138 |  |
| Body | JO | 7 | 7.13 | 6 | 0.000383 | 0.001** |  |
| Head | JO | 7 | 4.23 | 6 | 0.005 | 0.016* |  |
| **5-HT_7B_** | |  |  |  |  |  |  |
| **Tissue 1** | **Tissue 2** | **Sample size** | **Statistic** | **df** | **p value** | **Adj p value** |  |
| Body | Head | 7 | 1.57 | 6 | 0.169 | 0.507 |  |
| Body | JO | 7 | 3.91 | 6 | 0.008 | 0.024* |  |
| Head | JO | 7 | 3.88 | 6 | 0.008 | 0.024* |  |

**Supplemental Table 3: Statistical comparisons for male single injection data and median change per group**

Statistical comparisons (calculated using ART ANOVA) for changes in frequency for each single injection type for male *Ae. aegypti*, as well as the estimated median shift in frequency following injection. Stars indicate significance level (** p<0.01, *** p<0.001).

| **Group 1** | **Group 2** | **Estimate** | **SE** | **df** | **t ratio** | **Adj p value** |
| --- | --- | --- | --- | --- | --- | --- |
| 25 mM 5-HTP | 25 mM AMTP | 34.242 | 2.857 | 52 | 11.986 | 1.25E-15*** |
| 25 mM 5-HTP | 25 mM Serotonin | -3.991 | 3.1444 | 52 | -1.269 | 0.21002 |
| 25 mM 5-HTP | Ringer | 23.009 | 3.1444 | 52 | 7.317 | 9.26E-09*** |
| 25 mM 5-HTP | 5 mM Serotonin | 8.091 | 3.069 | 52 | 2.637 | 0.02203* |
| 25 mM AMTP | 25 mM Serotonin | -38.233 | 2.938 | 52 | -13.013 | 5.52E-17*** |
| 25 mM AMTP | Ringer | -11.233 | 2.938 | 52 | -3.823 | 0.00133** |
| 25 mM AMTP | 5 mM Serotonin | -26.152 | 2.857 | 52 | -9.154 | 1.61E-11*** |
| 25 mM Serotonin | Ringer | 27.000 | 3.218 | 52 | 8.389 | 2.17E-10*** |
| 25 mM Serotonin | 5 mM Serotonin | 12.082 | 3.144 | 52 | 3.842 | 0.00133** |
| Ringer | 5 mM Serotonin | -14.918 | 3.144 | 52 | -4.744 | 8.37E-05*** |

| **Injection type** | **Median change (Hz)** |
| --- | --- |
| 25 mM 5HTP | 130.426 |
| 25 mM AMTP | -66.274 |
| Ringer | -4.376 |
| 5 mM Serotonin | 75.537 |
| 25 mM Serotonin | 155.373 |

**Supplemental Table 4: Statistical comparisons for female single injection data and median change per group**

Statistical comparisons (calculated using ART ANOVA) for changes in frequency for each single injection type for female *Ae. aegypti*, as well as the estimated median shift in frequency following injection. Stars indicate significance level (* p<0.05).

| **Group 1** | **Group 2** | **Estimate** | **SE** | **df** | **t ratio** | **p value** |
| --- | --- | --- | --- | --- | --- | --- |
| Ringer | 5 mM Serotonin | -5.6 | 2.376 | 18 | -2.356 | 0.0299* |

| **Injection type** | **Median change (Hz)** |
| --- | --- |
| Ringer | -2.281 |
| 5 mM Serotonin | 7.789 |

**Supplemental Table 5: Statistical comparisons for male two injection data and median change per group**

Statistical comparisons (calculated using ART ANOVA) for changes in frequency after 25 mM serotonin injection, and then a second injection, for male *Ae. aegypti*, as well as the estimated median shift in frequency following each injection. Stars indicate significance level (* p<0.05).

**After 25 mM Serotonin injection**

| **Group 1** | **Group 2** | **Estimate** | **SE** | **df** | **t ratio** | **p value** |
| --- | --- | --- | --- | --- | --- | --- |
|  |  |  |  |  |  |  |
| 25 mM Serotonin + Ringer | 25 mM Serotonin +  5 mM Methiothepin | -1.6 | 2.692 | 18 | -0.594 | 0.560 |

| **Injection type** | **Median change (Hz)** |
| --- | --- |
| 25 mM Serotonin + Ringer | 128.390 |
| 25 mM Serotonin + 5 mM Methiothepin | 164.248 |

**After second injection**

| **Group 1** | **Group 2** | **Estimate** | **SE** | **df** | **t ratio** | **p value** |
| --- | --- | --- | --- | --- | --- | --- |
|  |  |  |  |  |  |  |
| 25 mM Serotonin + Ringer | 25 mM Serotonin +  5 mM Methiothepin | 5.6 | 2.376 | 18 | 2.356 | 0.0299* |

| **Injection type** | **Median change (Hz)** |
| --- | --- |
| 25 mM Serotonin + Ringer | -43.034 |
| 25 mM Serotonin + 5 mM Methiothepin | -69.948 |

**Supplemental Table 6: Statistical comparisons for male phonotaxis AUC data**

Statistical comparisons (calculated using Wilcoxon signed rank tests) for changes in AUC for male *Ae. aegypti* phonotaxis experiments after exposure to Ringer- or 5 mM AMTP-doped food. Stars indicate significance level (** p<0.01).

| **Group 1** | **Group 2** | **Sample size 1** | **Sample size 2** | **Median change** | **p value** |
| --- | --- | --- | --- | --- | --- |
| **Glucose / Ringer** |  |  |  |  |  |
| Glucose | Ringer | 10 | 10 | -5.138 | 0.6953 |
|  |  |  |  |  |  |
| **Glucose / AMTP** |  |  |  |  |  |
| Glucose | AMTP | 11 | 11 | -61.130 | 0.00195** |

**Supplemental Table 7: Statistical comparisons for male phonotaxis boundary frequency data and median frequency values**

Statistical comparisons (calculated using Wilcoxon signed rank tests) for changes in the range of frequencies to which male *Ae. aegypti* respond before/after exposure to Ringer or 5 mM AMTP-doped food, as well as estimated median values for upper and lower boundary frequencies. Stars indicate significance level (* p<0.05).

| **Group 1** | **Group 2** | **Sample size 1** | **Sample size 2** | **p value** |
| --- | --- | --- | --- | --- |
| **Glucose / Ringer** |  |  |  |  |
| Glucose | Ringer | 10 | 10 | 0.1309 |
|  |  |  |  |  |
| **Glucose / AMTP** |  |  |  |  |
| Glucose | AMTP | 11 | 11 | 0.0407* |

| **Experiment type** | **Median lower boundary frequency (Hz)** | **Median upper boundary frequency (Hz)** |
| --- | --- | --- |
| **Glucose / Ringer** |  |  |
| Glucose | 404.271 | 636.432 |
| Ringer | 413.317 | 604.271 |
|  |  |  |
| **Glucose / AMTP** |  |  |
| Glucose | 386.181 | 629.397 |
| AMTP | 400.251 | 587.186 |

**Supplemental Table 8: Median values for AUC and frequency bounds for male phonotaxis experiments including both AMTP and Ringer exposure**

Estimated median values for AUC and frequency boundaries for male *Ae. aegypti* phonotaxis experiments after exposure to glucose, 5 mM AMTP and then Ringer.

**AUC data**

| **Food type** | **Median values** |
| --- | --- |
|  |  |
| Glucose | 112.153 |
| AMTP | 57.343 |
| Ringer | 99.325 |

**Upper Frequency bound**

| **Food type** | **Median lower boundary frequency (Hz)** | **Median upper boundary frequency (Hz)** |
| --- | --- | --- |
|  |  |  |
| Glucose | 390.201 | 637.437 |
| AMTP | 402.261 | 587.186 |
| Ringer | 398.241 | 623.367 |
